# Integrated Metabolic and Inflammatory Clustering Reveals Distinct Risk Profiles for Digestive Diseases

**DOI:** 10.1002/advs.202511000

**Published:** 2025-09-28

**Authors:** Zhenhe Jin, Qichen Chen, Liangfeng Zhou, Kexin Ye, Zhaoxue Liu, Wenxi Jiang, Linwen Luo, Yize Wang, Xiaohua Ye, Chaohui Yu, Zhe Shen

**Affiliations:** ^1^ Department of Gastroenterology The First Affiliated Hospital School of Medicine Zhejiang University Hangzhou Zhejiang 310003 China; ^2^ Department of Gastroenterology Affiliated Jinhua Hospital Zhejiang University School of Medicine Jinhua Zhejiang 321000 China

**Keywords:** cluster analysis, digestive diseases, machine learning, metabolomics

## Abstract

Emerging research highlights the complex relationship between metabolic dysfunction and chronic low‐grade inflammation, which disrupts gut homeostasis and drives disease progression. However, most current studies evaluate metabolic and inflammatory markers separately, relying on basic indicators such as body mass index (BMI) or individual biomarkers. In this study, a scalable clustering framework is developed to integrate six clinical parameters in 398 432 participants from the UK Biobank, identifying four distinct metabolic‐inflammatory subtypes. Cox proportional hazards models demonstrate significant associations between these subtypes and digestive disease risk. Using 251 plasma metabolites and elastic net regression, cluster‐associated metabolite signatures are identified. Mediation analyses indicate that metabolic signatures mediate the association between clusters and digestive disease risk. Machine learning algorithms are applied to construct disease‐specific metabolic risk scores, achieving C‐indices above 0.70 for ten digestive disease endpoints. Explainable machine learning approaches further identify both shared and disease‐specific predictors, with glycoprotein acetyls, valine, tyrosine, and fatty acids emerging as key risk indicators. This integrative approach provides a comprehensive framework for digestive disease risk assessment and offers novel insights into the metabolic mechanisms underlying disease susceptibility.

## Graphical abstract

This study analyzes 398 432 participants, identifying four distinct metabolic‐inflammatory subtypes. These subtypes show a significant association with digestive disease risk. Cluster‐associated metabolite signatures partially explain this link. Machine learning models using these metabolites accurately predict risk for ten digestive diseases. Key risk indicators include glycoprotein acetyls, amino acids (valine, tyrosine), and fatty acids, revealing new mechanisms for disease susceptibility.

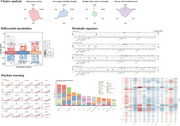

## 1 Introduction

Obesity represents a complex, multifaceted disorder that has escalated into a global epidemic, now impacting nearly half of the adult population worldwide.^[^ ^1^ ^]^ Beyond excessive body weight, obesity involves profound disruptions in metabolic and inflammatory homeostasis.^[^ ^2^, ^3^ ^]^ Adipose tissue, an active endocrine organ, secretes adipokines and pro‐inflammatory cytokines, linking metabolic dysfunction to persistent low‐grade inflammation.^[^ ^4^ ^]^ These intertwined pathways create a self‐perpetuating cycle where metabolic disturbances aggravate inflammation, while chronic inflammation impairs metabolic regulation.^[^ ^5^ ^]^ This reciprocal relationship, termed metaflammation, drives the development of metabolic diseases, including diabetes, non‐alcoholic fatty liver disease (NAFLD), and cardiovascular disease.^[^ ^6^ ^]^

Given the integral role of this metabolic‐inflammation interplay in systemic disease, increasing attention has turned to its implications for digestive health. The gastrointestinal tract, serving as a critical interface between environmental exposures and host metabolism, is highly responsive to both metabolic derangements and inflammatory stressors.^[^ ^7^ ^]^ Such interactions compromise mucosal integrity, reshape the gut microbiome, and facilitate malignant transformation. Accumulating evidence demonstrated that metabolic syndrome and chronic inflammation substantially elevate the risk and severity of NAFLD, inflammatory bowel disease (IBD), and gastrointestinal cancers.^[^ ^6^, ^8^, ^9^, ^10^ ^]^ Indeed, large‐scale analyses revealed metabolic syndrome more than doubles major liver complication risk and increases digestive malignancy incidence.^[^ ^11^ ^]^ Moreover, individuals with obesity frequently exhibit more aggressive digestive disease phenotypes and poorer outcomes,^[^ ^12^, ^13^ ^]^ highlighting the need to disentangle the distinct contributions of metabolic and inflammatory dysfunction in gastrointestinal pathology.

Despite these insights, most prior research has assessed metabolic and inflammatory markers separately, often relying on conventional anthropometric measures such as body mass index (BMI) for risk stratification.^[^ ^12^, ^13^ ^]^ This approach overlooks considerable heterogeneity, as individuals with similar BMI may present markedly different metabolic profiles and clinical trajectories.^[^ ^14^, ^15^ ^]^ Consequently, the combined effects of metabolic and inflammatory imbalances on digestive disease risk, along with the relevant molecular mechanisms, remain insufficiently characterized.

To address these limitations, we employed an integrative, data‐driven methodology to jointly characterize metabolic and inflammatory phenotypes, aiming to refine metabolic subtyping and enhance risk prediction for digestive diseases. Leveraging data from the UK Biobank, we developed a scalable clustering framework incorporating metabolic and inflammatory biomarkers and systematically compared the incidence of gastrointestinal disorders across subtypes. For each cluster, we identified distinctive metabolites using limma analysis and identified metabolomic signatures based on 251 plasma metabolites. Subsequently, we constructed disease‐specific metabolic risk scores (MetRS) using machine learning algorithms. This multidimensional strategy provides deeper mechanistic insight into the metabolic‐inflammation interplay that shapes digestive disease pathogenesis, and lays the groundwork for precision prevention and targeted therapeutic intervention.

## 2 Results

### 2.1 Clustering Characteristics

**Figure 1:**
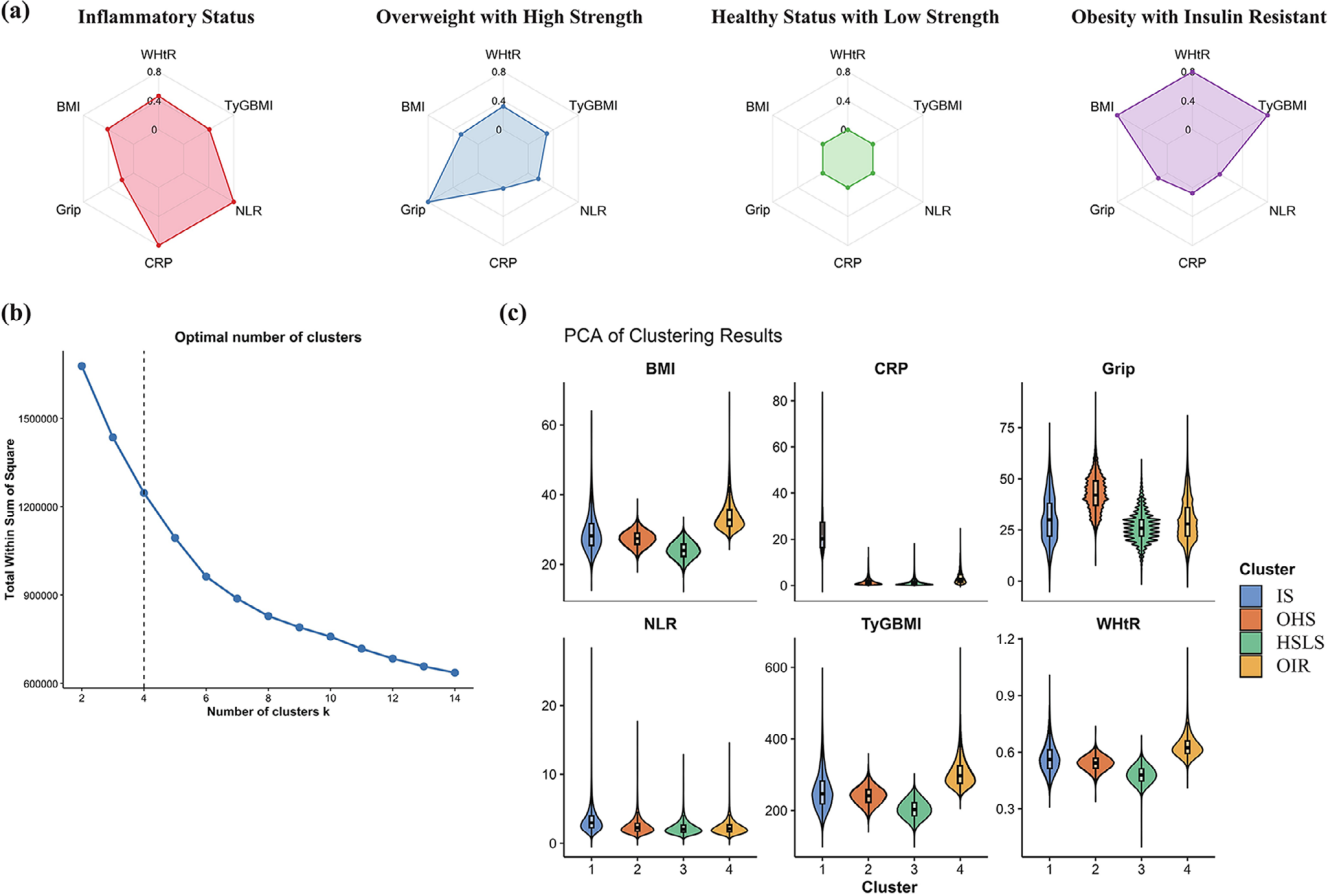
The proportion and features of different clusters in the cohort study. A) Radar plots summarize scaled variables for each cluster. Values closer to the outer ring are higher than the cohort average for each of the key variables. B) Elbow plot for determining the optimal number of clusters. C)The violin plot of the feature variables of different clusters. Abbreviations: BMI, body mass index; CRP, C‐reactive protein; NLR, Neutrophil‐to‐Lymphocyte Ratio; TyGBMI, TyG index multiplied by BMI; WHtR, Waist‐to‐Height Ratio; IS, inflammatory status; OHS, overweight with high strength; HSLS, healthy status with low strength; OIR, obesity with insulin resistance.

Six parameters related to metabolic disruption and inflammation were selected: body mass index (BMI), waist‐to‐height ratio (WHtR), grip strength, C‐reactive protein (CRP), neutrophil‐to‐lymphocyte ratio (NLR), and triglyceride‐glucose index‐BMI (TyG‐BMI). Each of these six parameters was significantly associated with the risk of developing almost all the 35 digestive diseases (Table S1, Supporting Information). As demonstrated in **Figure** **1**, four clusters emerged as optimal, with Jaccard similarity coefficients exceeding 0.80, confirming robust clustering stability. Furthermore, subgroup clustering analyses were performed by stratifying the population according to ethnicity and age. In all subgroups, four clusters consistently provided the best model fit (Figure S2, Supporting Information).

The analysis yielded four distinct anthropometric clusters: Cluster 1, designated as “healthy status with low strength” (HSLS), exhibited the lowest values across all parameters; Cluster 2, characterized by elevated CRP and NLR values, was classified as “inflammatory status” (IS); Cluster 3, distinguished by superior grip strength alongside moderately elevated BMI and WHtR, was termed “overweight with high strength” (OHS); Cluster 4, presenting with maximum BMI, WHtR, and TyG‐BMI values, was identified as “obesity with insulin resistance” (OIR).

**Table** **1** presents the baseline characteristics of these anthropometric clusters. The participant cohort had a mean age of 56.5 ± 8.1 years, with females comprising 53.8% of the study population.

**Table 1:** Basic characteristics of participants according to cluster subtype in the UK Biobank cohort.

	Healthy status with low strength (*N* = 168470)	Inflammatory status (*N* = 8173)	Overweight with high strength (*N* = 134512)	Obesity with insulin resistant (*N* = 87277)	Overall (*N* = 398432)	*p* value
**Basic characteristics**						
Sex						<0.001
Female	138401 (82.2%)	4399 (53.8%)	18803 (14.0%)	52907 (60.6%)	214510 (53.8%)	
Male	30069 (17.8%)	3774 (46.2%)	115709 (86.0%)	34370 (39.4%)	183922 (46.2%)	
Age						<0.001
Mean (SD), years	56.3 (8.10)	58.2 (7.92)	55.9 (8.24)	57.7 (7.68)	56.5 (8.09)	
Ethnicity						<0.001
White	159229 (94.5%)	7736 (94.7%)	127743 (95.0%)	81461 (93.3%)	376169 (94.4%)	
Other	9241 (5.5%)	437 (5.3%)	6769 (5.0%)	5816 (6.7%)	22263 (5.6%)	
TDI						<0.001
Mean (SD)	−1.51 (2.97)	−0.768 (3.32)	−1.54 (2.99)	−0.774 (3.26)	−1.34 (3.07)	
Education level						<0.001
College and above	60504 (35.9%)	1977 (24.2%)	46743 (34.8%)	20170 (23.1%)	129394 (32.5%)	
High school and below	107966 (64.1%)	6196 (75.8%)	87769 (65.3%)	67107 (76.9%)	269038 (67.5%)	
**Lifestyle**						
Alcohol drinking						<0.001
Never	7754 (4.6%)	452 (5.5%)	3461 (2.6%)	5457 (6.3%)	17124 (4.3%)	
Previous	5391 (3.2%)	486 (5.9%)	3805 (2.8%)	4177 (4.8%)	13859 (3.5%)	
Current	155325 (92.2%)	7235 (88.5%)	127246 (94.6%)	77643 (89.0%)	367449 (92.2%)	
Smoking						<0.001
Never	101260 (60.1%)	3776 (46.2%)	69076 (51.4%)	45036 (51.6%)	219148 (55.0%)	
Previous	50956 (30.2%)	3121 (38.2%)	50075 (37.2%)	33915 (38.9%)	138067 (34.7%)	
Current	16254 (9.6%)	1276 (15.6%)	15361 (11.4%)	8326 (9.5%)	41217 (10.3%)	
Physical activity						<0.001
Low	19388 (11.5%)	1571 (19.2%)	18882 (14.0%)	16628 (19.1%)	56469 (14.2%)	
Moderate	54598 (32.4%)	2428 (29.7%)	42916 (31.9%)	25735 (29.5%)	125677 (31.5%)	
High	56451 (33.5%)	1951 (23.9%)	48530 (36.1%)	20232 (23.2%)	127164 (31.9%)	
Missing	38033 (22.6%)	2223 (27.2%)	24184 (18.0%)	24682 (28.3%)	89122 (22.4%)	
**Baseline comorbidity**						
Hypertension, n [%]	28257 (16.8%)	2936 (35.9%)	36403 (27.1%)	37921 (43.4%)	105517 (26.5%)	<0.001
Hyperlipidemia, n [%]	29599 (17.6%)	2385 (29.2%)	36951 (27.5%)	32062 (36.7%)	100997 (25.3%)	<0.001
Diabetes, n [%]	2995 (1.8%)	588 (7.2%)	5595 (4.2%)	10680 (12.2%)	19858 (5.0%)	<0.001
**Drug history**						
Aspirin use, n [%]	15036 (8.9%)	1394 (17.1%)	20872 (15.5%)	17634 (20.2%)	54936 (13.8%)	<0.001
No aspirin NSAIDs, n [%]	51094 (30.3%)	3132 (38.3%)	32386 (24.1%)	31765 (36.4%)	118377 (29.7%)	<0.001
Hypolipidemic drug, n [%]	12190 (7.2%)	614 (7.5%)	2047 (1.5%)	11953 (13.7%)	26804 (6.7%)	<0.001
**Metabolic and inflammatory markers**						
WHtR						<0.001
Mean (SD)	0.480 (0.0455)	0.568 (0.0796)	0.541 (0.0389)	0.631 (0.0563)	0.535 (0.0744)	
BMI [kg m^−2^]						<0.001
Mean (SD)	24.0 (2.48)	29.1 (5.58)	27.4 (2.28)	33.8 (4.08)	27.4 (4.75)	
Grip strength (kg)						<0.001
Mean (SD)	26.6 (7.29)	30.4 (11.5)	43.0 (8.75)	29.5 (10.3)	32.8 (11.3)	
CRP [mg L^−1^]						<0.001
Mean (SD)	1.49 (1.75)	24.0 (11.7)	1.80 (1.85)	3.74 (3.14)	2.55 (4.23)	
NLR						<0.001
Mean (SD)	2.21 (0.875)	3.36 (1.78)	2.44 (1.03)	2.26 (0.887)	2.32 (0.975)	
TyG‐BMI						<0.001
Mean (SD)	204 (25.8)	256 (55.3)	241 (25.5)	306 (40.8)	240 (49.4)	

Abbreviations: BMI, body mass index; CRP, C‐reactive protein; NLR, neutrophil‐to‐lymphocyte ratio; SD, standard deviation; TDI, Townsend deprivation index; TyG, triglyceride‐glucose index; WHtR, waist‐to‐height ratio.

### 2.2 Digestive Diseases Risk of Anthropometric Clusters

**Figure 2:**
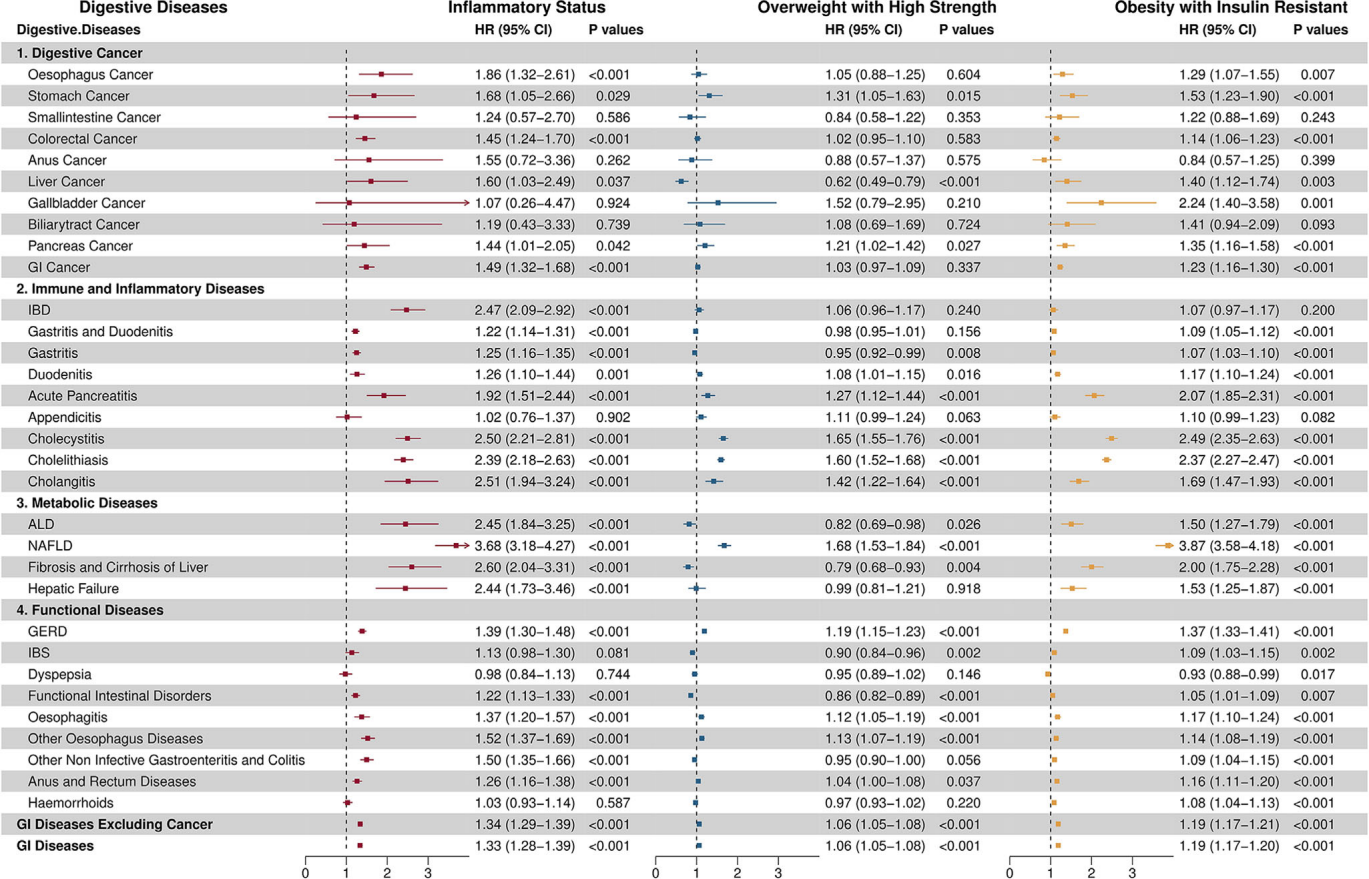
Cox analysis of the associations of anthropometric clusters with risks of digestive diseases, compared to Healthy Status with Low Strength, respectively. Model adjusted for age, age squared, sex, ethnicity, Townsend deprivation index, smoking status, alcohol drinking, education level, physical activity, medicinal intake (aspirin, non‐aspirin NSAIDs, and lipid‐lowering drugs use), and comorbidities (lipidaemia, hypertension, diabetes).

During a median follow‐up period of 12.8 years, 125718 individuals developed digestive diseases. **Figure** **2** illustrates the differential incidence rates of digestive diseases across the anthropometric clusters. The IS cluster demonstrated significantly elevated risk for multiple endpoints compared to HSLS: five types of digestive cancers, eight immune and inflammatory diseases, four metabolic diseases, and six functional disorders, with hazard ratios (HRs) ranging from 1.22 to 3.68. Moreover, as demonstrated in Figure S4 (Supporting Information), participants categorized in the IS cluster exhibited the highest cumulative rates of digestive diseases spanning multiple categories: three digestive cancers, five immune and inflammatory diseases, three metabolic diseases, and four functional disorders.

The OHS cluster presented the lowest cumulative incidence rates for irritable bowel syndrome (IBS), dyspepsia, and functional intestinal disorders among all clusters, while demonstrating elevated risk for two digestive cancers, five immune and inflammatory diseases, one metabolic disease, and four functional disorders compared to HSLS (HRs: 1.04‐1.68). Notably, this cluster demonstrated reduced risk for liver cancer, gastritis, alcoholic liver disease (ALD), hepatic fibrosis and cirrhosis, IBS, dyspepsia, and functional intestinal disorders.

The OIR cluster displayed the highest cumulative incidence rates for gallbladder cancer, NAFLD, dyspepsia, and hemorrhoids. This cluster exhibited elevated risk for six digestive cancers, seven immune and inflammatory diseases, four metabolic diseases, and nine functional disorders compared to HSLS (HRs: 1.05–3.87). Both the IS and OIR clusters demonstrated comparably elevated risks for acute pancreatitis, cholecystitis, cholelithiasis, and gastroesophageal reflux disease (GERD).

### 2.3 Subgroup and Sensitive Analyses

Subgroup analyses stratified by age (with 45 years as the cutoff), sex, and ethnicity showed that, for the majority of digestive diseases, the associations between anthropometric clusters and disease risk maintained broadly consistent across different subgroups (Tables S2–S4, Supporting Information). To investigate the impact of hereditary predispositions, the polygenic risk score (PRS) for obesity was included in the subgroup analysis. The results showed that, for most digestive diseases, there was no significant difference in incidence among individuals with different genetic predispositions (Table S5, Supporting Information).

In our sensitivity analyses, additional adjustment for familial cancer history was implemented. Furthermore, when excluding individuals diagnosed within two years of recruitment or those with less than two years of follow‐up, the results remained consistent. These modified analyses yielded results that remained concordant with our primary findings, thus substantiating the robustness of the observed associations (Tables S6–S8, Supporting Information).

### 2.4 Differential Metabolites of Anthropometric Clusters

**Figure 3:**
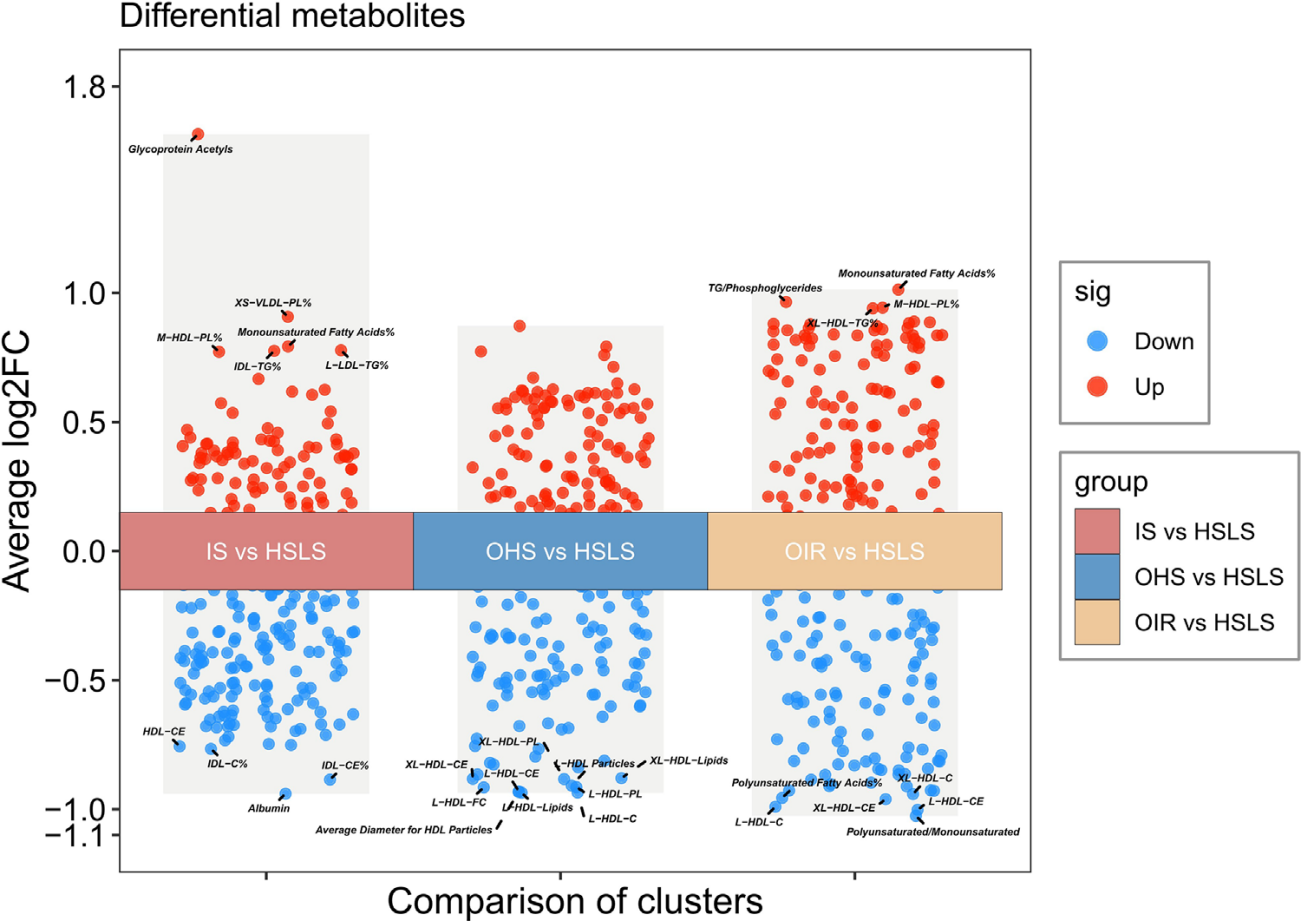
Volcano diagrams showing the differential metabolites across clusters. The red dot on the figure represents the upregulated metabolite, and the blue dot represents the downregulated metabolite. The *y*‐axis corresponds to log2 (fold change).

A limma analysis was performed on 251 metabolites to examine the differential metabolite profiles between HSLS and other clusters. As shown in **Figure** **3**, glycoprotein acetyls (GlycA) were found to be significantly more abundant in Cluster IS, whereas albumin, cholesteryl esters to total lipids in IDL percentage (IDL‐CE%), cholesterol to total lipids in IDL percentage (IDL‐C%), and cholesteryl esters in HDL (HDL‐CE) showed significantly reduced abundance. In Cluster OHS, HDL‐related metabolites exhibited significantly lower abundance. Cluster OIR had higher levels of monounsaturated fatty acids, but lower levels of polyunsaturated fatty acids and HDL‐related metabolites.

For KEGG analysis, we selected metabolites with significant abundance differences across all three clusters. The KEGG pathways revealed important processes in the synthesis and breakdown of valine, leucine, and isoleucine, as well as phenylalanine metabolism (Figure S5, Supporting Information).

### 2.5 Identification of the Cluster‐ Associated Metabolic Signatures

**Figure 4:**
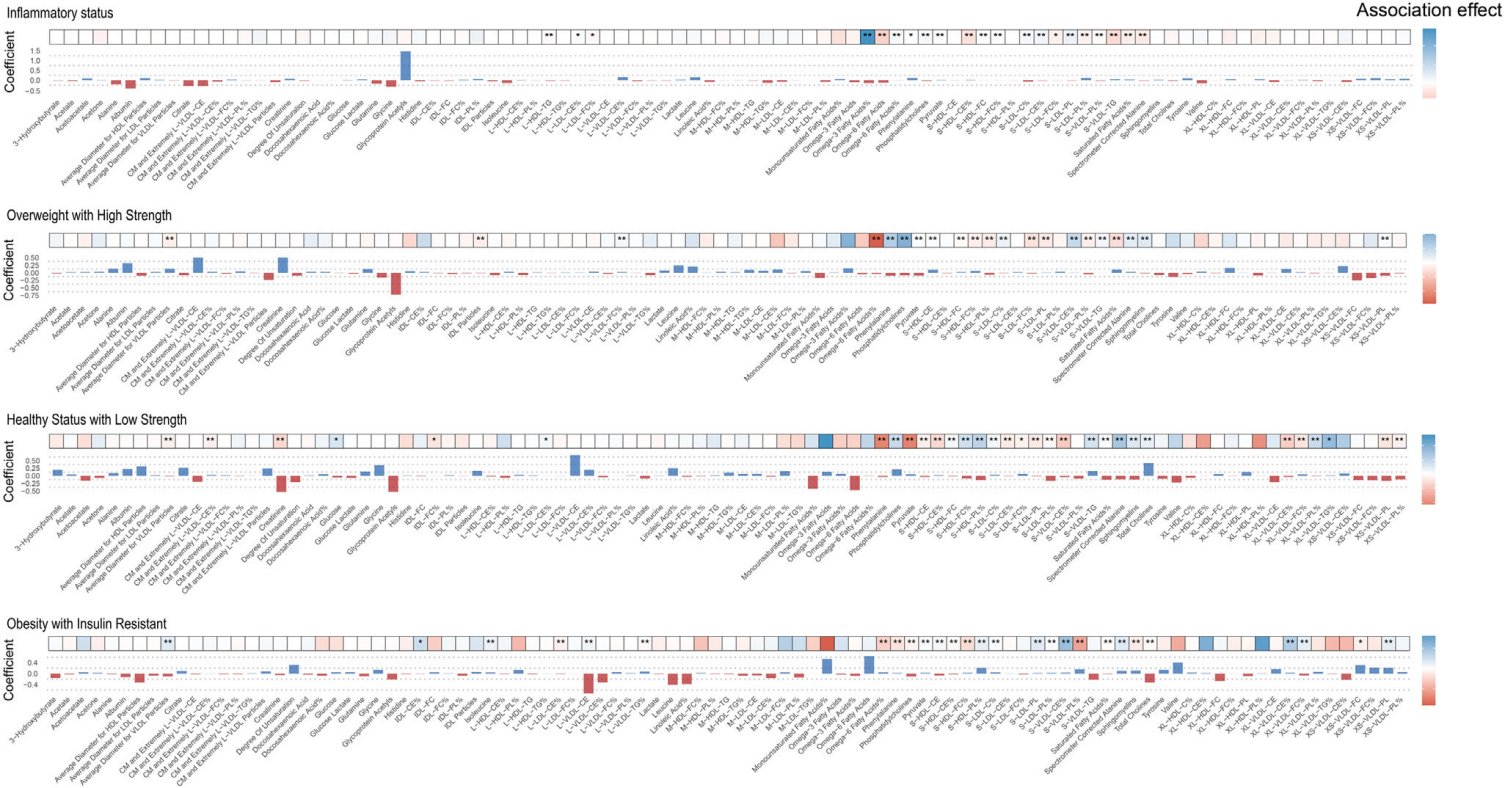
Associations between selected metabolites constituting the metabolic signature and four clusters. Each panel presented the associations between metabolites in the metabolic signature and relevant cluster. Colors indicate the direction of association, with red representing positive associations and blue indicating inverse associations. The darkness of the color corresponds to the magnitude of the association. Asterisks denote the significance level of associations (^*^ *p* < 0.05 and Bonferroni‐corrected *p* < 0.05). Abbreviations: C, cholesterol; CE, cholesteryl ester; FC, free cholesterol; M, medium; PL, phospholipid; TG, triglyceride; S, small; L, large; VLDL, very LDL; XL, very large; XS, very small; XXL, especially large.

Elastic net regression identified cluster‐associated metabolite profiles constituting the overall metabolic signatures. The analysis yielded 97, 96, 94, and 97 metabolites for the HSLS, IS, OHS, and OIR clusters, respectively (**Figure** **4**). Notably, 91 metabolites demonstrated consistent associations across all four clusters. The metabolic signatures predominantly comprised lipids, fatty acids, triglycerides, lipoproteins, amino acids, and ketone bodies, along with metabolites implicated in inflammatory processes, glycolytic pathways, and fluid homeostasis.

Mediation analyses elucidated the mechanistic pathways linking clusters to digestive disease risk (Tables S9–S11, Supporting Information). The metabolic signatures demonstrated significant mediating effects in the associations between all three non‐reference clusters and multiple endpoints, including GERD, gastritis, duodenitis, NAFLD, cholecystitis, cholelithiasis, cholangitis, and acute pancreatitis. Additionally, the metabolic signatures mediated the relationships between the IS cluster and IBD, non‐infective gastroenteritis and colitis, ALD, and hepatic failure. Analogous mediating effects were observed for the relationships between the OIR cluster and oesophagitis, non‐infective gastroenteritis and colitis, ALD, and hepatic failure.

### 2.6 MetRS Stratifies the Risk of Multiple Diseases

**Figure 5:**
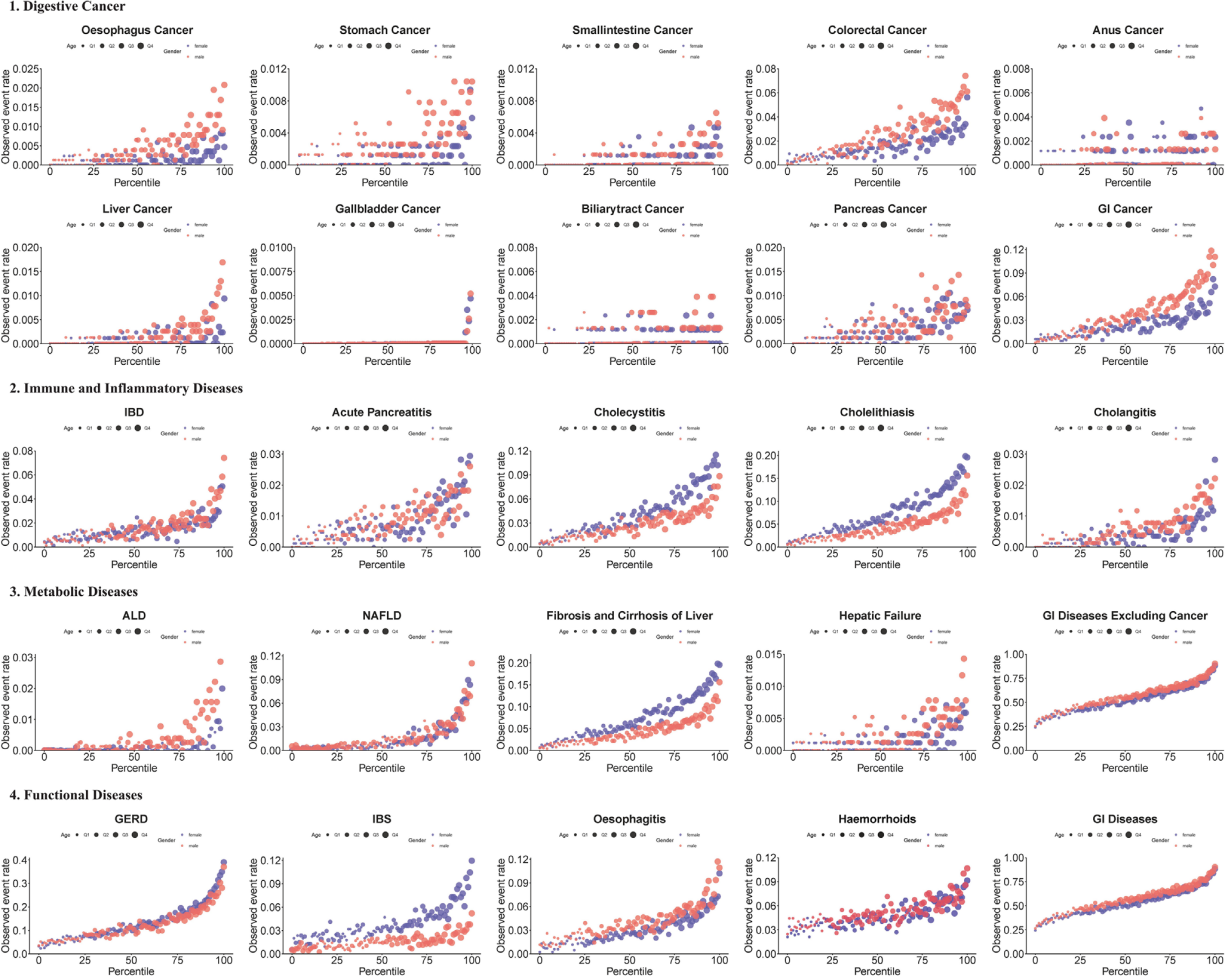
Observed event frequency for 25 incident disease categories plotted against best model percentiles. Age is represented by varying sizes of dots, while sex is distinguished by different colors of the dots.

We selected a subset of digestive diseases with higher clinical incidence rates for ML (LightGBM, XGBoost, Elastic Net Regression, and Random Forest). The metabolites, anthropometric clusters, and covariates were used as predictors for these diseases. Each model was used to predict each disease individually, and the best model was selected based on the ROC curve (Figure S6, Supporting Information). The model demonstrated robust predictive performance across various endpoints, particularly liver conditions such as ALD, NAFLD, liver cancer, liver fibrosis, cirrhosis, and hepatic failure. Higher MetRS correlated with increased event rates for specific digestive diseases (**Figure** **5**). Gender differences were observed in risk levels. Females had a higher risk of cholecystitis, cholelithiasis, liver fibrosis and cirrhosis, GERD, and IBS. In contrast, males demonstrated a higher risk of esophageal cancer, colorectal cancer, digestive cancer, ALD, and oesophagitis.

### 2.7 Contributions of Metabolites to Prediction Across Digestive Disease Spectrum

**Figure 6:**
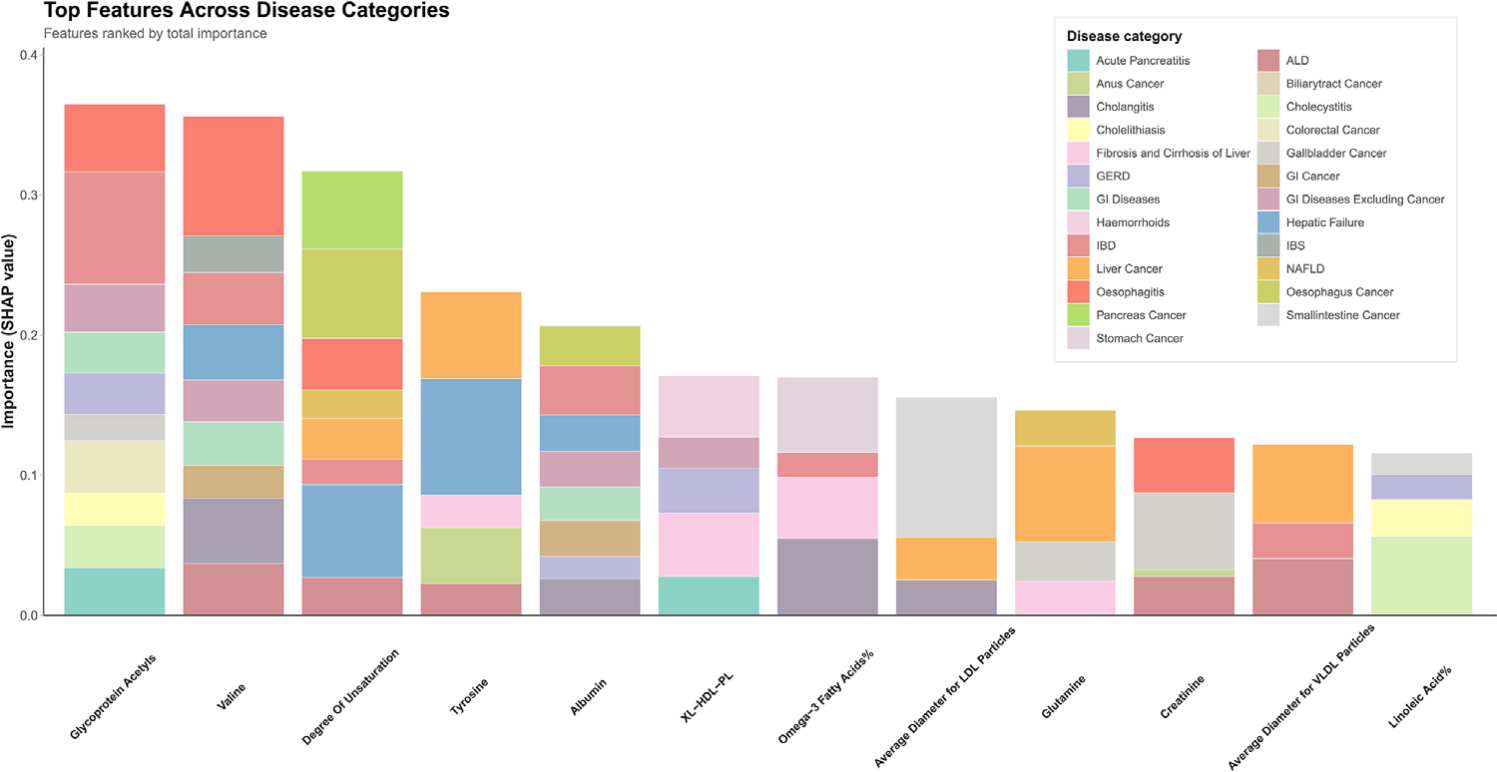
Stacked bar chart of standardized SHAP values from machine learning models across 25 disease categories. We highlighted 12 metabolites that exhibited the most important discriminatory value in three or more disease categories. Abbreviations: SHAP SHapley Additive exPlanations.

**Figure 7:**
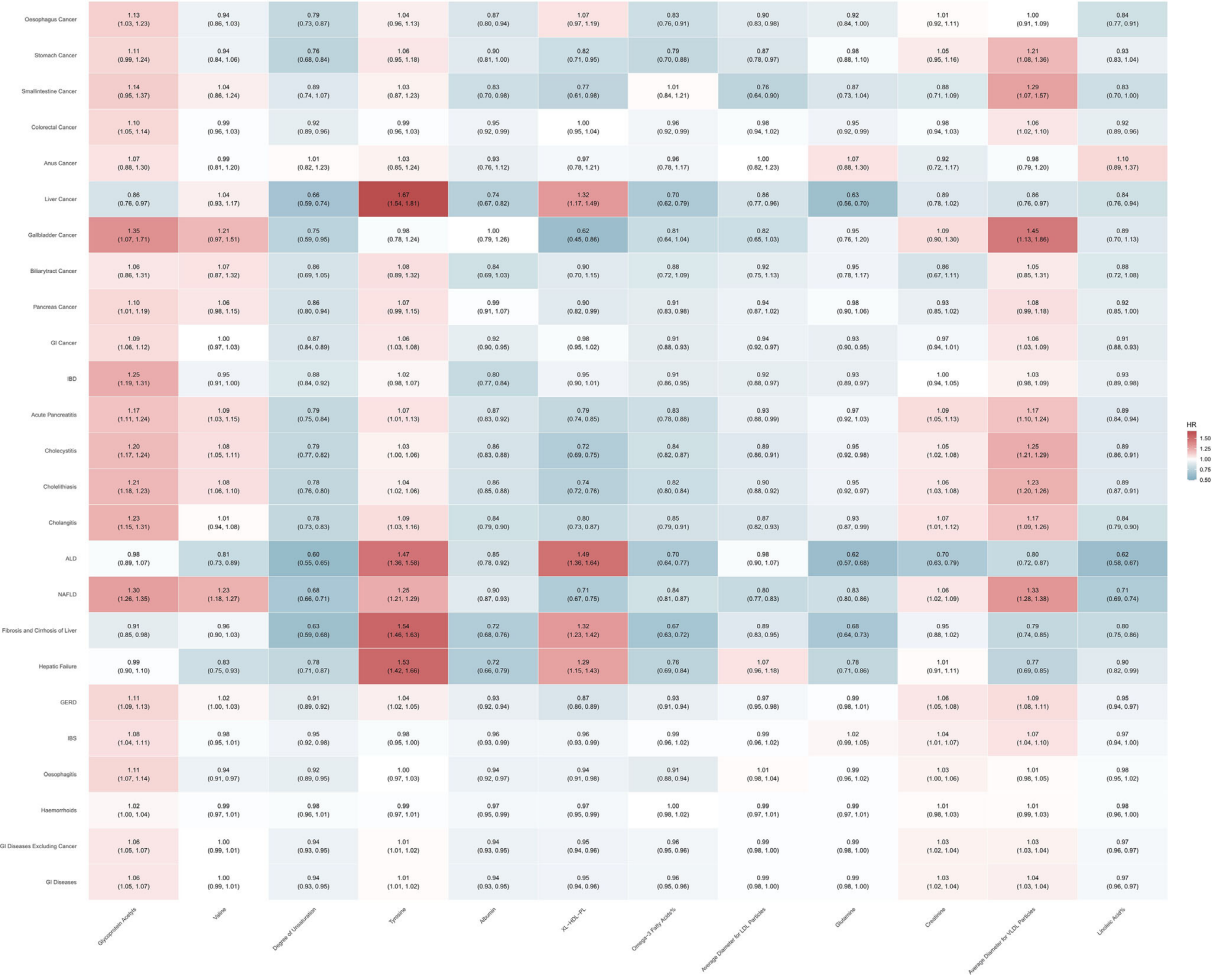
Associations between metabolites shown in Figure 6 and 25 outcomes. In each square, the first row presents the HR values from the Cox analysis, while the second row indicates the corresponding 95% confidence intervals. The color of squares indicates the effect size (HR). Model adjusted for age, age squared, sex, ethnicity, Townsend deprivation index, smoking status, alcohol drinking, education level, physical activity, medicinal intake (aspirin, non‐aspirin NSAIDs, and lipid‐lowering drugs use), and comorbidities (lipidaemia, hypertension, diabetes). Abbreviations: HR, hazard ratio.

We ranked the metabolites according to their predictive importance for various endpoints by leveraging SHAP values. Consequently, we identified the most influential discriminators linked to each condition (**Figure** **6**). Several metabolites demonstrated consistent predictive utility across multiple digestive pathologies, including glycoprotein acetyls, valine, degree of unsaturation of fatty acids, tyrosine, albumin, phospholipids in very large HDL (XL‐HDL‐PL), omega‐3 fatty acids%, average diameter for LDL particles, glutamine, creatinine, average diameter for VLDL particles, and linoleic acid%. Notably, GlycA, valine, and the degree of unsaturation of fatty acids emerged as particularly robust predictors, demonstrating significant discriminative capacity across 10, 9, and 8 categories of digestive diseases, respectively. In addition, these metabolites were also significantly associated with the risk of these digestive diseases (**Figure** **7**).

GlycA and the average diameter of VLDL particles were associated with an increased risk of most of these diseases. In contrast, higher unsaturation of fatty acids, albumin levels, omega‐3 fatty acids%, average LDL particle diameter, glutamine, and linoleic acid% were linked to a lower risk of these diseases. Specifically, tyrosine was associated with a higher risk of liver diseases, including liver cancer, ALD, NAFLD, liver fibrosis, cirrhosis, and hepatic failure.

### 2.8 Functional Annotation of Immune Proteins Correlated with Key Metabolites

To further investigate the relationship between metabolism and immune regulation, Pearson correlation analyses were performed between the key predictive metabolites and immune‐related plasma proteins. Proteins that showed significant correlations with these metabolites were selected for downstream functional annotation.

GO enrichment analysis revealed that the significantly associated proteins are primarily involved in immune receptor activity, cytokine binding, cell adhesion, enzyme inhibitor activity, chemokine activity, and a range of immune‐related biological processes, including chemotaxis, leukocyte migration, and the regulation of inflammatory responses (Figure S7, Supporting Information). Similarly, KEGG pathway enrichment analysis demonstrated that these proteins are enriched in multiple immune and inflammation‐related pathways, such as cytokine‐cytokine receptor interaction, chemokine signaling, NF‐kappa B signaling, Toll‐like receptor signaling, and pathways linked to infection and autoimmune diseases (Figures S8, Supporting Information).

These findings indicate that the immune‐related proteins most strongly associated with key metabolites are functionally involved in various immune and inflammatory mechanisms, suggesting a potential mediating role in disease pathogenesis.

## 3 Discussion

In this study, we utilized a data‐driven clustering approach to enhance the classification of anthropometric‐metabolic subtypes within the UK Biobank cohort. Six routinely available clinical parameters enabled the identification of four distinct clusters, which were systematically analyzed for associations with digestive disease risk. The analytical framework integrated differential metabolite profiling, KEGG pathway enrichment, and mediation analyses, followed by machine learning modeling to evaluate the predictive value of key metabolites for digestive diseases.

Our findings indicated that individuals with obesity (mean BMI 33.4 kg m^−^^2^) and insulin resistance had a higher risk of developing various digestive diseases compared to the reference cluster characterized by healthy status and low grip strength. This finding was in accordance with prior studies that have demonstrated correlations between obesity, insulin resistance, and digestive diseases.^[^ ^16^, ^17^, ^18^ ^]^ Conversely, overweight individuals (BMI 27.4 kg m^−^^2^) with high grip strength showed characteristics similar to the reference cluster and were associated with a lower risk of liver cancer, ALD, and liver fibrosis/cirrhosis. Similarly, a case‐control study reported no significant association between overweight status and the risk of HCC among metabolically healthy individuals.^[^ ^19^ ^]^ In contrast, Korean population data showed that metabolically healthy obese individuals still had an elevated risk of liver cirrhosis.^[^ ^20^ ^]^ These differing observations may be influenced by sample size, ethnicity, and population heterogeneity. Furthermore, our study found that inflammatory status clusters faced higher digestive diseases risk, consistent with research linking elevated inflammatory markers to increased risks of IBD, liver diseases, and digestive tract cancers.^[^ ^21^, ^22^, ^23^ ^]^ Notably, previous studies using only BMI often miss the complexity of metabolic health.^[^ ^12^, ^13^ ^]^ By combining anthropometric, metabolic, and inflammatory markers, our approach enables more precise risk stratification and offers new insights beyond conventional BMI‐based assessment.

In metabolomic analyses, limma analysis and subsequent KEGG pathway analysis revealed that the major metabolic distinctions among clusters were concentrated in amino acid metabolism, particularly the biosynthesis, degradation, and metabolic processing of valine, leucine, isoleucine, phenylalanine, tyrosine, and tryptophan. Elevated levels of branched‐chain amino acids (BCAAs), such as valine, leucine, and isoleucine, are associated with obesity and insulin resistance.^[^ ^24^ ^]^ Prolonged consumption of high‐BCAA diets has been linked to an increased risk of obesity and a shortened lifespan.^[^ ^25^ ^]^ Additionally, aromatic amino acids, including phenylalanine, tyrosine, and tryptophan, have also been reported to be associated with obesity and insulin resistance.^[^ ^26^ ^]^ Further analysis identified distinct metabolic signatures associated with each anthropometric‐metabolic cluster and demonstrated their mediating role in the pathway from cluster assignment to the risk of various digestive diseases. Previous studies have demonstrated that metabolites served as key actors in ALD, NAFLD, IBD, acute pancreatitis, and gallbladder and biliary tract diseases.^[^ ^27^, ^28^, ^29^, ^30^, ^31^ ^]^ Furthermore, metabolic signatures were shown to mediate the association between clusters and GERD, consistent with findings from a study in children with GERD, where alterations in tyrosine metabolism, glycine, serine, and threonine metabolism were reported.^[^ ^32^ ^]^ The potential mediating role of metabolic signatures pointed to promising avenues for both intervention and risk prediction. Targeting key metabolic pathways through interventions such as lifestyle modification, pharmacological treatment, or dietary changes may help reduce the risk of digestive diseases in high‐risk subgroups. Future research should evaluate, in large population‐based cohorts, whether metabolic interventions can effectively improve digestive health outcomes. Incorporating metabolic profiling into clinical risk models can improve the early identification of high‐risk individuals, allowing for timelier and tailored preventive strategies.

The integration of machine learning methodologies enabled us to harness the predictive potential of the identified metabolic signatures. By constructing disease‐specific metabolic risk scores, we demonstrated strong predictive value across a spectrum of digestive diseases, particularly for metabolic conditions such as fatty liver disease, fibrosis, cirrhosis, and hepatic failure. Among the most influential discriminators were GlycA, amino acids, and fatty acids, with each showing significant associations with disease risk across multiple endpoints. Notably, many of these metabolites overlapped with those identified in our earlier differential metabolite analysis and KEGG pathway enrichment, underscoring the robustness and biological relevance of our findings. GlycA was notably linked to increased liver disease and colorectal cancer risk, consistent with previous evidence showing higher incidence and mortality of colorectal cancer (HR per SD = 1.26, 95% CI: 1.15–1.39).^[^ ^33^ ^]^ GlycA also emerges as a promising IBD activity biomarker, with 9–20% increased IBS risk.^[^ ^34^, ^35^ ^]^ Amino acid metabolism also played an important role in disease prediction. Our analysis revealed that valine and tyrosine were significantly associated with liver disease risk, in agreement with previous studies demonstrating elevated serum valine as an independent risk factor for NAFLD (OR = 1.13, 95% CI: 1.04–1.23),^[^ ^36^ ^]^ and increased tyrosine as a marker of disease progression.^[^ ^37^ ^]^ With regard to fatty acids, our results showed an inverse association between polyunsaturated fatty acids and risk for most liver diseases, consistent with cohort evidence that n‐3 and n‐6 polyunsaturated fatty acids are protective, whereas saturated fatty acids increase the risk of hepatocellular carcinoma and chronic liver disease mortality.^[^ ^38^ ^]^ In IBD, saturated fatty acids and ω‐6 polyunsaturated fatty acids have been associated with pro‐inflammatory effects, whereas ω‐3 polyunsaturated fatty acids exhibit anti‐inflammatory properties. Lipid mediators derived from polyunsaturated fatty acids play important roles in modulating immune responses and maintaining gut barrier integrity.^[^ ^39^ ^]^ In IBS and other functional gastrointestinal disorders, alterations in polyunsaturated fatty acids, particularly n‐3 and n‐6 PUFAs, have been observed compared to healthy individuals. These changes are thought to influence gut inflammation, barrier function, and visceral sensitivity, thereby contributing to symptom development in IBS.^[^ ^40^ ^]^ Taken together, these findings underscore the value of integrating metabolic profiling with machine learning for more precise risk stratification and deeper mechanistic understanding of both metabolic and immune‐mediated digestive diseases.

An important advantage of this study is that all six parameters (BMI, WHtR, grip strength, CRP, NLR, and TyG‐BMI) are routinely available in clinical settings. These measures require no specialized equipment or advanced testing and can be readily obtained in both primary and secondary care. Their accessibility makes this clustering framework highly practical for risk stratification in real‐world settings, including those with limited resources. By relying solely on standard clinical assessments, the approach enables clinicians to identify individuals at increased risk for digestive diseases and provides straightforward early warning signs to support timely and informed decision‐making. This adaptability across diverse healthcare environments underscores the framework's potential utility in both well‐resourced and resource‐constrained contexts.

Beyond the clinical setting, these findings have significant public health implications. The ability to delineate distinct metabolic‐inflammatory subtypes in a large, population‐based cohort underscores the heterogeneity of digestive disease risk in the general population. This knowledge can inform the development of more nuanced public health policies, guide targeted prevention initiatives, and improve the allocation of screening resources. For instance, integrating these routinely collected indicators into population health programs may enhance early detection and intervention efforts, ultimately reducing the burden of digestive diseases.

Correlation analysis shed light on the interplay between metabolic status and immune regulation. Functional annotation of immune‐related proteins revealed significant enrichment in pathways linked to cytokine and chemokine signaling, immune receptor activity, and leukocyte migration. Notably, pathways such as cytokine‐cytokine receptor interactions, which were most prominently enriched, were well‐established contributors to the pathogenesis of inflammatory bowel disease, liver disorders, and gastrointestinal cancers.^[^ ^41^, ^42^, ^43^ ^]^ These findings suggested that changes in metabolic profiles might have influenced immune responses via these molecular pathways. This metabolic‐immune interaction likely helped explain the heterogeneity in digestive disease risk across clinical subtypes. Clinically, integrating metabolic and immunologic profiling might have improved risk assessment and identified new therapeutic targets. Future studies should clarify the causal relationships involved.

This study has several limitations. First, our clustering approach assigned each participant to a single metabolic subtype, which may not fully capture the continuous or overlapping variation in metabolic and inflammatory profiles among individuals. Second, although we employed five‐fold cross‐validation and repeated assessment data to mitigate overfitting and enhance the robustness of our findings, these internal validation measures do not fully eliminate the risk of bias. Ideally, validation in an independent cohort would be necessary to more accurately assess the generalizability of our results across diverse populations. Third, as the UK Biobank cohort is predominantly composed of healthy, White European volunteers, the applicability of our findings to other ethnicities or populations with different health backgrounds may be limited. Additionally, potentially important factors such as genetic background, environmental exposures, and gut microbiota were not included in our analysis. Future research should validate these findings in more heterogeneous and multi‐ethnic populations and integrate multi‐omics data to further refine disease risk stratification and mechanistic understanding.

In conclusion, our data‐driven approach, based on six routinely accessible clinical measures, successfully delineated distinct anthropometric metabolic subtypes that demonstrate differential associations with digestive diseases. This multidimensional classification surpasses the conventional BMI‐based approach in capturing the complexity and heterogeneity of metabolic health. Furthermore, we identified key cluster‐specific metabolites and metabolic pathways, highlighted the mediating role of metabolic signatures, and demonstrated the substantial predictive value of specific metabolites in digestive disease risk stratification. These findings provide novel insights into the mechanisms connecting metabolic health and digestive pathology, offering valuable directions for targeted prevention, early intervention, and personalized management of digestive diseases.

## 4 Experimental Section

### Study Design and Participants

The UK Biobank is a large, ongoing cohort study that includes over half a million participants. Participants were recruited between 2006 and 2010 from 22 assessment centers located in England, Scotland, and Wales, with ages at baseline ranging from 40 to 69 years (https://www.ukbiobank.ac.uk/enable‐your‐research/about‐our‐data/baseline‐assessment).^[^ ^44^ ^]^ The following exclusion criteria were applied to this study: 1) withdrawal of consent; 2) absence of demographic information; 3) incomplete data on clustering variables; and 4) unavailable outcome data. The participant selection process is depicted in Figure S1 (Supporting Information).

### Plasma Metabolite Profiling

Plasma metabolite profiling performed at baseline spanned from June 2019 to April 2020. A total of 251 metabolic measures were quantified in each plasma sample using nuclear magnetic resonance (NMR) spectroscopy, consisting of 170 absolute levels and 81 derived ratios. The samples were drawn from a randomly selected subset of ≈ 280 000 UK Biobank participants. Detailed descriptions of the Nightingale Health NMR biomarker platform and experimental protocols are described elsewhere.^[^ ^45^ ^]^ In this study, metabolomics data at baseline were utilized, with all metabolic biomarkers being logarithmically transformed and standardized before analysis.

### Plasma Proteomic Assessment

Blood samples collected at baseline (2007–2010) were analyzed using the Olink Explore Proximity Extension Assay, an antibody‐based platform that enables high‐throughput and standardized quantification of plasma proteins. Proteins exhibiting more than 30% missing data were omitted from the analysis. All remaining protein concentrations were normalized using Z‐score transformation prior to downstream statistical evaluation. Pearson correlation analyses were used to assess associations between metabolites and immune‐related proteins. Proteins with significant correlations underwent GO and KEGG pathway enrichment analyses.

### Clinical Outcomes

All participants were prospectively followed until death, loss to follow‐up, or censorship, with the end dates varying by region: 31 October 2022 for England, 31 May 2022 for Wales, and 31 August 2022 for Scotland. Health outcome data for UK Biobank participants were primarily derived from linkage to hospital inpatient records and cancer registries. The International Classification of Diseases, Tenth Revision (ICD‐10) was employed to identify cases of digestive diseases, encompassing a total of 30 distinct types, which included 9 types of digestive cancers, 8 immune and inflammatory disorders, 4 metabolic diseases, and 9 functional disorders, as detailed in Table S12 (Supporting Information).

### Covariates

At recruitment, covariate data were collected through touchscreen questionnaires and verbal interviews to address potential confounding biases. The variables adjusted for included age, sex, ethnicity, education level, Townsend Deprivation Index (TDI), smoking status, alcohol consumption, physical activity, medication use (e.g., aspirin, non‐aspirin NSAIDs, lipid‐lowering drugs), and comorbidities such as hypertension, hyperlipidaemia, and diabetes. In the primary analysis, categorical variables with ≥5% missing values were excluded, while those with <5% missing values and continuous variables were imputed using the most frequent category and the median, respectively.

### Clustering Analysis

In this study, objectively measurable metabolic and inflammatory markers, including body mass index (BMI), waist‐to‐height ratio (WHtR), grip strength, C‐reactive protein (CRP), neutrophil‐to‐lymphocyte ratio (NLR), and triglyceride‐glucose index‐BMI (TyG‐BMI) were selected to serve as cluster variables. These markers were chosen due to their accessibility and clinical relevance as indicators of general obesity, abdominal obesity, fitness, insulin resistance, and inflammation. K‐means clustering was performed,^[^ ^46^ ^]^ and the elbow method was used to determine the optimal number of clusters (Figure 1). To assess cluster stability, the Jaccard coefficient was calculated using the bootstrap method (*n* = 100). T‐distributed stochastic neighbor embedding (t‐SNE) was employed to visualize the distribution of the distinct clusters (Figure S3, Supporting Information).

### Machine Learning Models

Four machine learning (ML) techniques were implemented to predict multi‐endpoints onset: LightGBM, XGBoost, Elastic Net logistic regression, and Random Forest. The model was developed using a dataset split into training (80%) and testing (20%) sets. Advanced feature engineering was conducted, including handling of missing values, extreme value winsorization (1st‐99th percentiles), feature transformations, and interaction term generation.

To address class imbalance, a customized Synthetic Minority Over‐sampling Technique (SMOTE) approach was employed targeting a positive‐to‐negative ratio of 0.3, with direct oversampling for extremely rare positive cases (*n* ≤ 5) and strategic undersampling for moderate imbalance situations. For model development, each algorithm was optimized with specific configurations. LightGBM employed Bayesian optimization for hyperparameter tuning, with a search space including number of leaves (7–511), learning rate (0.001–0.3), feature/bagging fractions (0.5–1.0), depth (3–20), and regularization terms, along with scale_pos_weight for class imbalance.^[^ ^47^ ^]^ XGBoost was configured with a binary logistic objective, AUC metric, and optimized subsample parameters.^[^ ^48^ ^]^ Elastic Net used fivefold cross‐validation with alpha = 0.5 to balance L1/L2 regularization.^[^ ^49^ ^]^ Random Forest was constructed with 500 trees and sqrt (features) split selection.^[^ ^50^ ^]^

Performance evaluation focused on the area under the receiver operating characteristic curve (AUC), with additional visualization of ROC curves across all models. The model that demonstrated the best performance was selected as the final one. In addition, feature importance was evaluated using SHAP (SHapley Additive exPlanations) values to identify and rank the most influential predictors.^[^ ^51^ ^]^ This approach quantified the contribution of each feature to individual predictions and allowed for global model interpretation.

To assess clinical utility, two complementary analyses were conducted: 1) Q‐Plot analysis, which stratified predictions into disease‐specific metabolic risk score (MetRS), calculated observed event rates within each percentile, and visualized the relationship between model predictions and actual outcomes with gender‐specific and age‐stratified assessments; and 2) Kaplan–Meier survival analysis, which categorized patients into risk tertiles (low, intermediate, high) based on predicted scores, enabling time‐to‐event analysis using follow‐up data and statistical comparison of disease‐free survival curves across risk groups.

Additionally, cross‐disease comparative analysis was performed to identify common metabolic signatures and disease‐specific predictive patterns. By aggregating feature importance metrics across multiple disease models, features that consistently appeared in top rankings (frequency ≥4 in top‐15 features) across different conditions were identified. This approach allowed us to explore potential shared metabolic pathways and biological mechanisms underlying various disease processes, while highlighting condition‐specific metabolic markers.

### Statistical Analyses

Continuous data were presented as mean (SD), and categorical variables were presented as frequency (percentage). Cox proportional hazards regression models were employed to estimate hazard ratios (HRs) and corresponding 95% confidence intervals (CIs) for the association between anthropometric clusters and digestive disease incidence. The models were adjusted for demographic factors (age, sex, ethnicity, education level, Townsend Deprivation Index), lifestyle variables (smoking status, alcohol consumption, physical activity), medication use (aspirin, non‐aspirin non‐steroidal anti‐inflammatory drugs, lipid‐lowering agents), and comorbidities (hypertension, lipidaemia, diabetes). Schoenfeld residuals testing confirmed the proportional hazards assumption (*p* > 0.05). Kaplan–Meier plots were generated for each endpoint across the four anthropometric clusters to visualize survival patterns. Additionally, stratified analyses were conducted based on key demographic and lifestyle factors. To assess robustness, sensitivity analyses included further adjustment for family history of cancer and exclusion of participants diagnosed within two years of recruitment or with insufficient follow‐up duration.

For metabolite differential abundance analysis, the limma approach (R package version 3.58.1) was implemented.^[^ ^52^ ^]^ Linear models were fitted for each metabolite using the lmFit function, followed by empirical Bayes smoothing of standard errors. Between‐group comparisons were quantified as log_2_ fold changes (logFC), and thresholds were established based on these values for subsequent screening.

To identify a specific metabolic signature associated with each anthropometric cluster, the baseline metabolomics data were partitioned into training (80%) and testing (20%) sets. An elastic net regression model was constructed to regress anthropometric clusters on 251 standardized metabolites. Optimal regularization parameters were determined through five‐fold cross‐validation, selecting the lambda value yielding a mean squared error within one standard error of the minimum. The composite metabolic signature was derived by calculating the weighted sum of selected metabolites, with weights corresponding to the regression coefficients. Model stability was subsequently evaluated on the testing set. To investigate potential mediating effects, mediation analyses were conducted using the CMAverse R package,^[^ ^53^ ^]^ applying a regression‐based approach with the Cox model to evaluate the role of metabolic signatures in the relationship between anthropometric clusters and digestive disease incidence.

Statistical analyses and graphical visualizations of results were conducted using R version 4.1.2. To correct for multiple comparisons across four groups with three comparisons per outcome, the Bonferroni correction was applied, with a significance threshold set at *p* < 0.05.

## Conflict of Interest

The authors declare no conflict of interest.

## Author Contributions

Z.J., X.Y., C.Y., and Z.S. contributed to the study concept and design; Z.J., X.Y., Q.C., L.Z., and K.Y. to the analysis and interpretation of the data; Z.J., X.Y., Z.L., W.J., L.L., and Y.W. to the drafting of the manuscript; and Z.J., X.Y., C.Y., and Z.S. to the critical revision of the manuscript.

## Supporting information



Supporting Information

## Data Availability

Data sharing is not applicable to this article as no new data were created or analyzed in this study.
