# Supplementary material for: Integrated Metabolic and Inflammatory Clustering Reveals Distinct Risk Profiles for Digestive Diseases
Source: Adv Sci (Weinh). 2025 Sep 28;12(44):e11000. doi: 10.1002/advs.202511000 (PMC12667462; doi:10.1002/advs.202511000)
Supplement: Supplementary file 1 — Supporting Information [file ADVS-12-e11000-s001.docx]

**Figure S1.** Participants selection flow diagram of UK Biobank cohort.


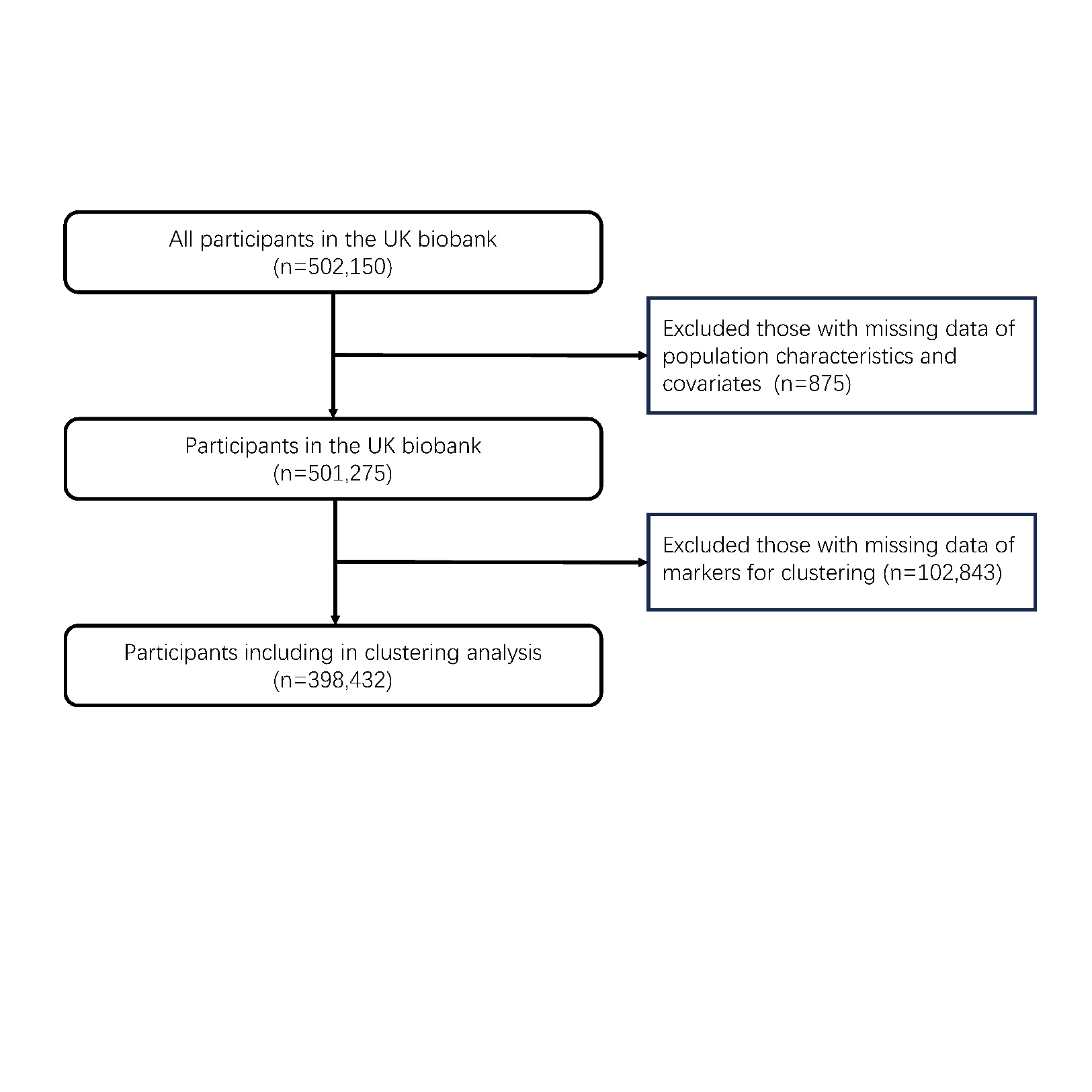


**Figure S2.** t-SNE visualization of the clustering data. Cluster 1: Inflammatory Status, Cluster 2: Overweight with High Strength, Cluster 3: Healthy Status with Low Strength, Cluster 4: Obesity with Insulin Resistant.

Abbreviations: t-SNE, t-Distributed Stochastic Neighbor Embedding.


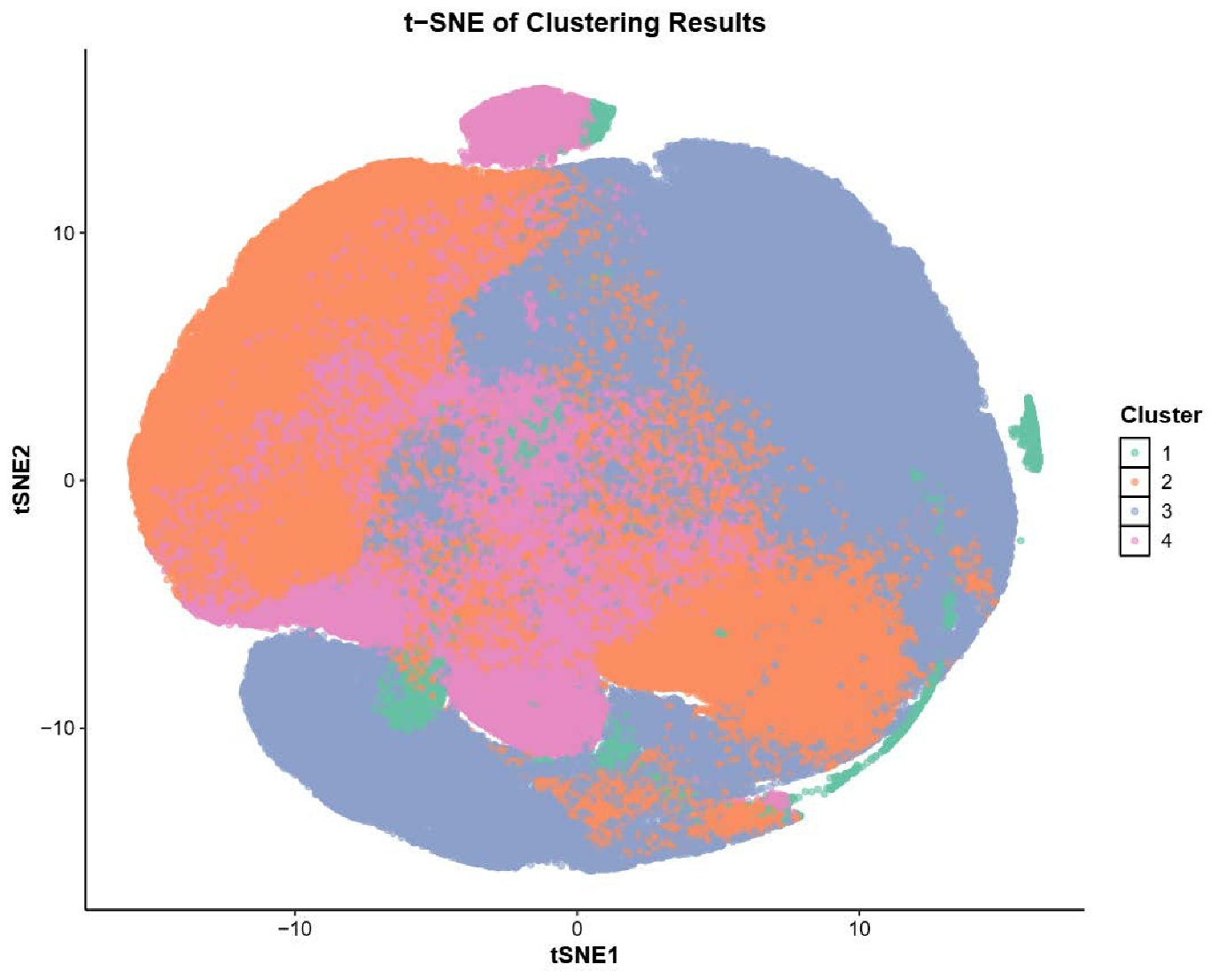


**Figure S3.** Kaplan–Meier curves of the association between cluster subtypes and risk of digestive diseases

Abbreviations: IS, Inflammatory Status; OHS, Overweight with High Strength; HSLS, Healthy Status with Low Strength; OIR, Obesity with Insulin Resistant.

**
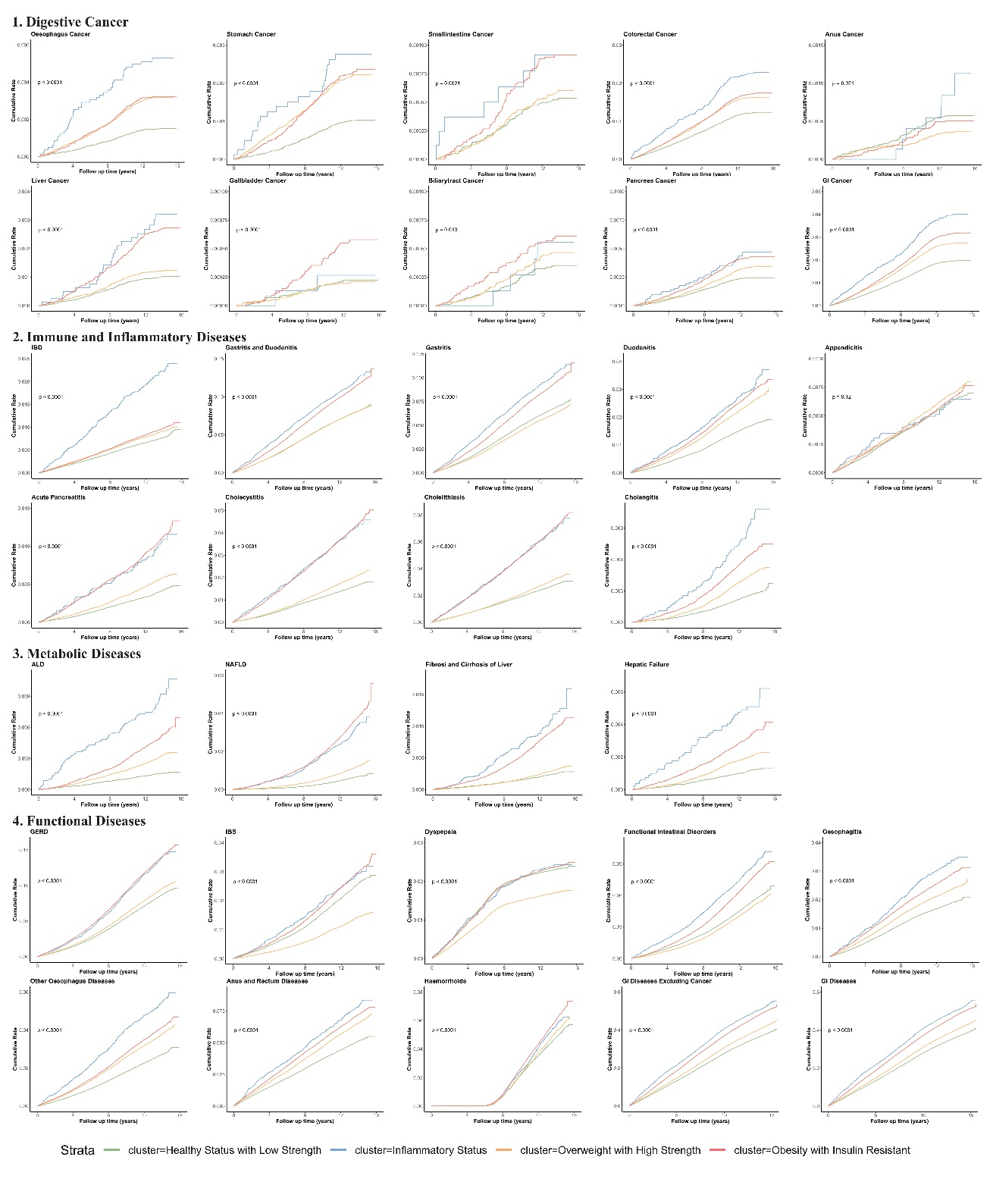
**

**Figure S4.** KEGG pathway analysis of differential metabolites identified by Limma analysis across four clusters.

**
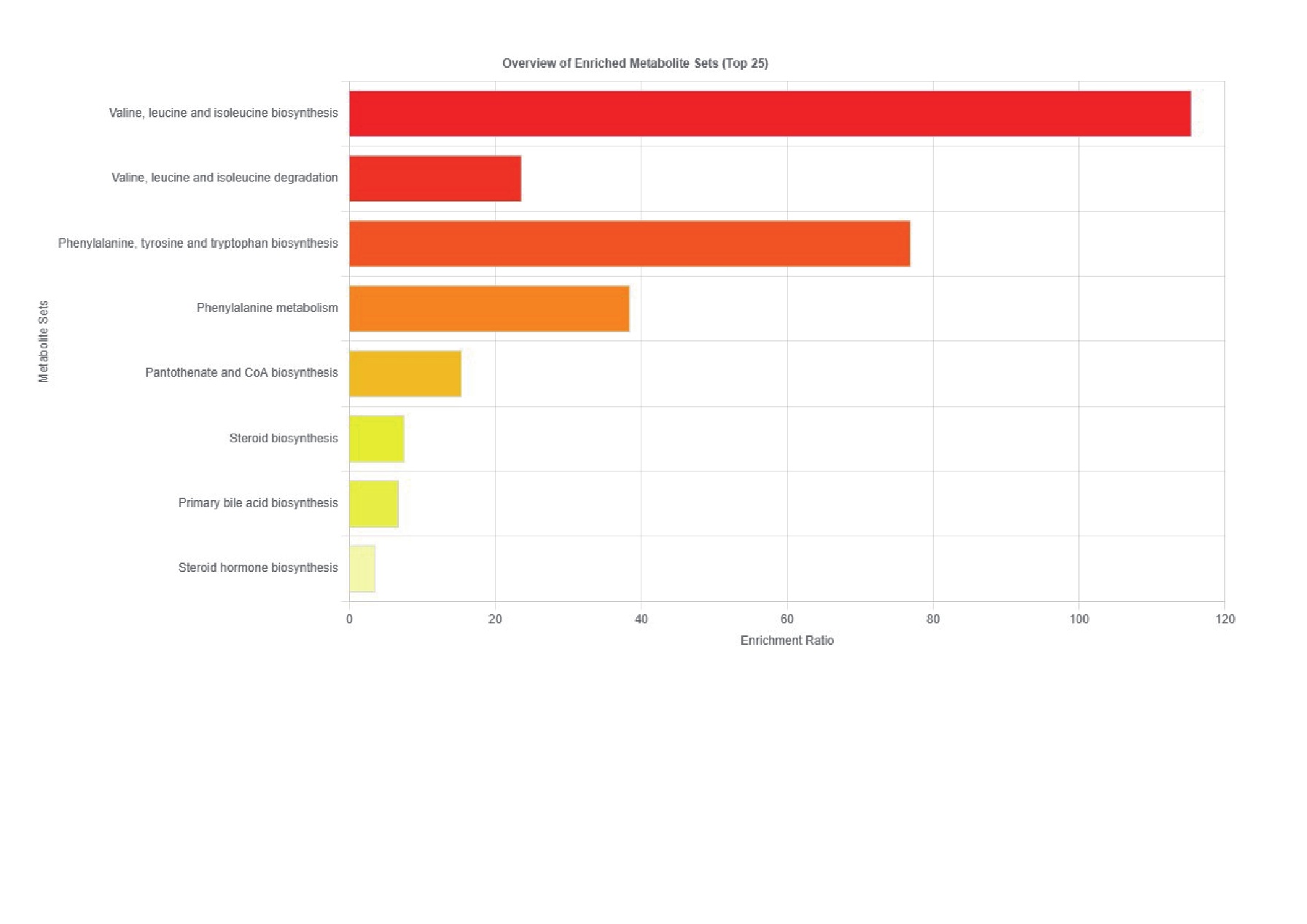
**

**Figure S5.** ROC curves of digestive diseases prediction models. For each disease, LightGBM, XGBoost, Elastic Net, and Random Forest were employed, and the model with the highest AUC value was selected.

Abbreviations: AUC, Area under the ROC Curve

**
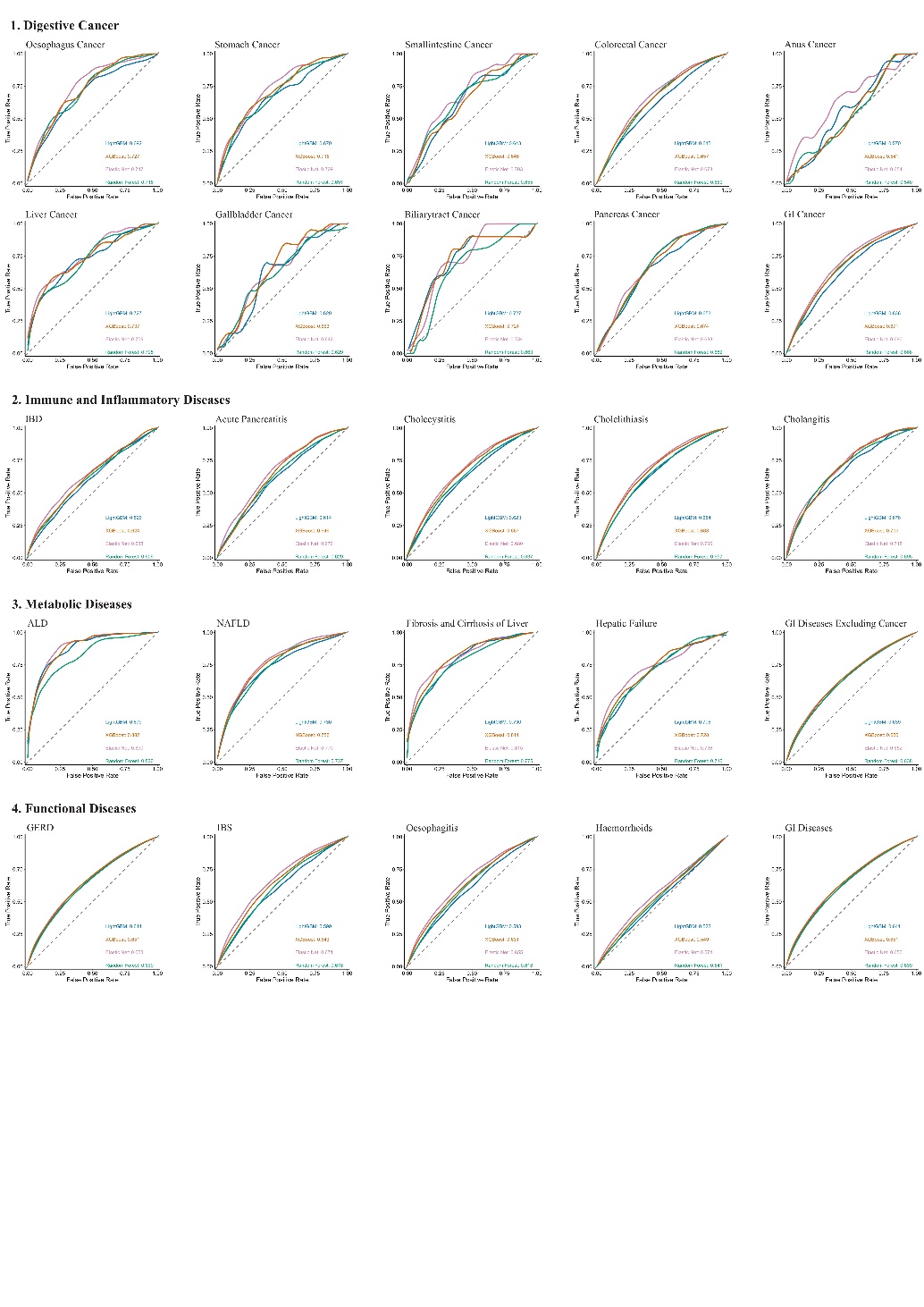
**

**Figure S6.** GO Enrichment and KEGG Pathway Analysis of Metabolite-Associated Immune Proteins.

Abbreviations: GO, Gene Ontology; KEGG, Kyoto Encyclopedia of Genes and Genomes.**
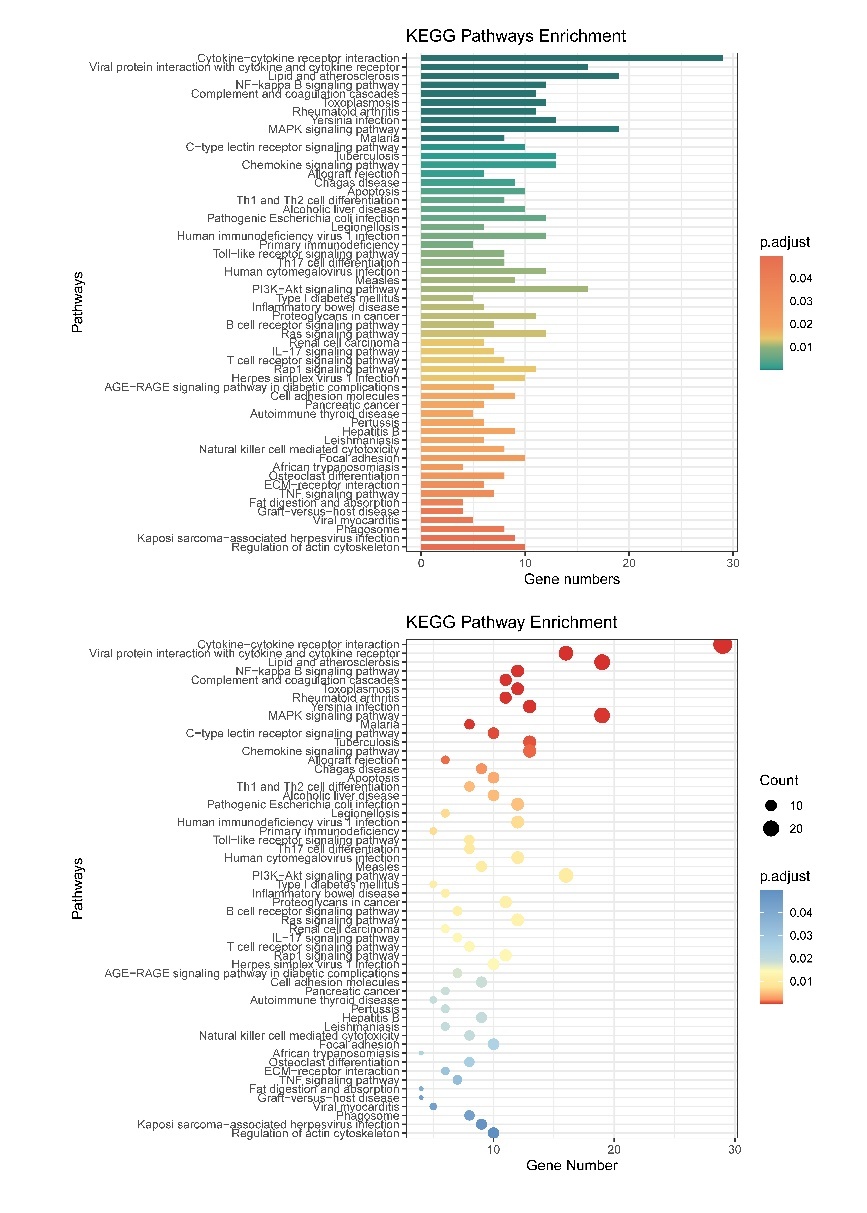

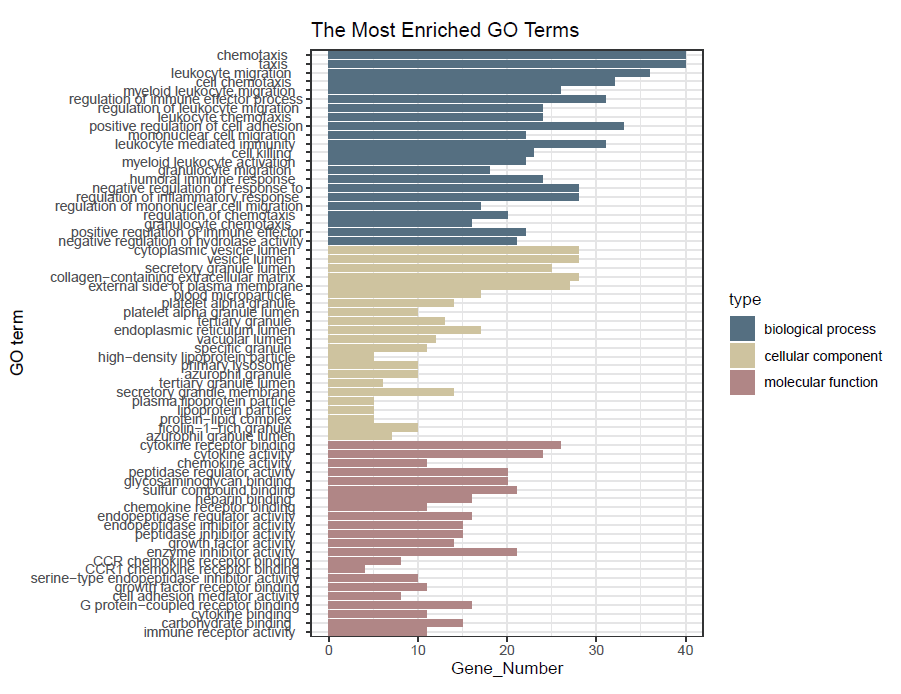
**

**Table S1.** Cox Regression Analysis of Six Clinical Parameters in Relation to the Risk of Digestive Diseases

| **Diseases** | **BMI** | | **WHtR** | | **Grip strength** | | **CRP** | | **NLR** | | **TyG-BMI** | |
| --- | --- | --- | --- | --- | --- | --- | --- | --- | --- | --- | --- | --- |
|  | **HR 95%CI** | ***P*** | **HR 95%CI** | ***P*** | **HR 95%CI** | ***P*** | **HR 95%CI** | ***P*** | **HR 95%CI** | ***P*** | **HR 95%CI** | ***P*** |
| **Oesophagus Cancer** | 1.03 (1.02, 1.05) | <0.001 | 14.06 (5.73, 34.49) | <0.001 | 1.00 (0.99, 1.01) | 0.970 | 1.02 (1.01, 1.03) | <0.001 | 1.10 (1.04, 1.17) | <0.001 | 1.00 (1.00, 1.00) | <0.001 |
| **Stomach Cancer** | 1.04 (1.02, 1.06) | <0.001 | 15.49 (5.34, 44.97) | <0.001 | 1.01 (1.00, 1.02) | 0.288 | 1.02 (1.00, 1.03) | 0.015 | 1.04 (0.97, 1.12) | 0.255 | 1.00 (1.00, 1.01) | <0.001 |
| **Smallintestine Cancer** | 1.03 (1.01, 1.06) | 0.014 | 5.77 (1.00, 33.26) | 0.050 | 0.99 (0.97, 1.01) | 0.271 | 1.02 (1.00, 1.04) | 0.027 | 1.03 (0.91, 1.16) | 0.690 | 1.00 (1.00, 1.01) | 0.006 |
| **Colorectal Cancer** | 1.02 (1.01, 1.02) | <0.001 | 3.37 (2.32, 4.90) | <0.001 | 1.00 (1.00, 1.00) | 0.523 | 1.02 (1.01, 1.02) | <0.001 | 1.04 (1.01, 1.06) | 0.006 | 1.00 (1.00, 1.00) | <0.001 |
| **Anus Cancer** | 0.97 (0.94, 1.01) | 0.116 | 0.35 (0.05, 2.71) | 0.317 | 0.98 (0.96, 1.00) | 0.088 | 1.04 (1.02, 1.06) | <0.001 | 1.00 (0.86, 1.16) | 0.978 | 1.00 (0.99, 1.00) | 0.091 |
| **Liver Cancer** | 1.06 (1.05, 1.08) | <0.001 | 119.73 (40.06, 357.86) | <0.001 | 0.97 (0.96, 0.98) | <0.001 | 1.03 (1.02, 1.04) | <0.001 | 0.98 (0.90, 1.06) | 0.595 | 1.01 (1.01, 1.01) | <0.001 |
| **Gallbladder Cancer** | 1.06 (1.03, 1.10) | <0.001 | 87.44 (8.82, 867.08) | <0.001 | 1.00 (0.97, 1.03) | 0.923 | 1.02 (1.00, 1.05) | 0.088 | 1.08 (0.91, 1.29) | 0.374 | 1.01 (1.00, 1.01) | <0.001 |
| **Biliarytract Cancer** | 1.03 (1.00, 1.07) | 0.039 | 4.49 (0.54, 37.42) | 0.165 | 0.99 (0.97, 1.01) | 0.294 | 1.00 (0.96, 1.03) | 0.930 | 1.04 (0.90, 1.20) | 0.607 | 1.00 (1.00, 1.01) | 0.017 |
| **Pancreas Cancer** | 1.04 (1.03, 1.05) | <0.001 | 11.32 (5.21, 24.58) | <0.001 | 1.00 (0.99, 1.01) | 0.937 | 1.01 (1.00, 1.02) | 0.018 | 1.07 (1.01, 1.13) | 0.017 | 1.00 (1.00, 1.01) | <0.001 |
| **GI Cancer** | 1.03 (1.02, 1.03) | <0.001 | 6.29 (4.74, 8.35) | <0.001 | 1.00 (1.00, 1.00) | 0.475 | 1.02 (1.01, 1.02) | <0.001 | 1.04 (1.02, 1.06) | <0.001 | 1.00 (1.00, 1.00) | <0.001 |
| **IBD** | 1.00 (1.00, 1.01) | 0.382 | 2.06 (1.28, 3.32) | 0.003 | 0.99 (0.98, 0.99) | <0.001 | 1.04 (1.03, 1.04) | <0.001 | 1.22 (1.19, 1.25) | <0.001 | 1.00 (1.00, 1.00) | 0.020 |
| **Gastritis and Duodenitis** | 1.02 (1.02, 1.02) | <0.001 | 5.29 (4.56, 6.13) | <0.001 | 0.98 (0.98, 0.99) | <0.001 | 1.01 (1.01, 1.02) | <0.001 | 1.05 (1.04, 1.06) | <0.001 | 1.00 (1.00, 1.00) | <0.001 |
| **Gastritis** | 1.02 (1.02, 1.02) | <0.001 | 4.76 (4.05, 5.59) | <0.001 | 0.98 (0.98, 0.99) | <0.001 | 1.01 (1.01, 1.02) | <0.001 | 1.05 (1.04, 1.06) | <0.001 | 1.00 (1.00, 1.00) | <0.001 |
| **Duodenitis** | 1.02 (1.02, 1.03) | <0.001 | 8.04 (6.02, 10.75) | <0.001 | 0.99 (0.98, 0.99) | <0.001 | 1.01 (1.01, 1.02) | <0.001 | 1.07 (1.05, 1.09) | <0.001 | 1.00 (1.00, 1.00) | <0.001 |
| **Acute Pancreatitis** | 1.07 (1.06, 1.08) | <0.001 | 119.21 (71.78, 198.00) | <0.001 | 0.99 (0.98, 1.00) | <0.001 | 1.03 (1.02, 1.03) | <0.001 | 1.07 (1.03, 1.12) | <0.001 | 1.01 (1.01, 1.01) | <0.001 |
| **Appendicitis** | 1.01 (1.00, 1.02) | 0.078 | 1.66 (0.96, 2.89) | 0.072 | 1.00 (1.00, 1.01) | 0.322 | 1.00 (0.99, 1.01) | 0.500 | 0.98 (0.94, 1.03) | 0.457 | 1.00 (1.00, 1.00) | 0.063 |
| **Cholecystitis** | 1.08 (1.08, 1.09) | <0.001 | 259.37 (201.66, 333.60) | <0.001 | 0.99 (0.99, 0.99) | <0.001 | 1.03 (1.03, 1.03) | <0.001 | 1.06 (1.03, 1.08) | <0.001 | 1.01 (1.01, 1.01) | <0.001 |
| **Cholelithiasis** | 1.08 (1.08, 1.08) | <0.001 | 206.85 (169.62, 252.26) | <0.001 | 0.99 (0.99, 0.99) | <0.001 | 1.03 (1.03, 1.03) | <0.001 | 1.06 (1.04, 1.07) | <0.001 | 1.01 (1.01, 1.01) | <0.001 |
| **Cholangitis** | 1.07 (1.06, 1.08) | <0.001 | 112.84 (60.30, 211.16) | <0.001 | 0.99 (0.98, 1.00) | 0.010 | 1.03 (1.03, 1.04) | <0.001 | 1.14 (1.10, 1.19) | <0.001 | 1.01 (1.01, 1.01) | <0.001 |
| **ALD** | 1.05 (1.04, 1.06) | <0.001 | 296.64 (142.80, 616.24) | <0.001 | 0.97 (0.96, 0.98) | <0.001 | 1.04 (1.03, 1.04) | <0.001 | 1.13 (1.07, 1.19) | <0.001 | 1.01 (1.00, 1.01) | <0.001 |
| **NAFLD** | 1.12 (1.11, 1.12) | <0.001 | 5635.74 (4221.82, 7523.20) | <0.001 | 0.98 (0.98, 0.98) | <0.001 | 1.04 (1.04, 1.04) | <0.001 | 0.99 (0.97, 1.02) | 0.556 | 1.01 (1.01, 1.01) | <0.001 |
| **Fibrosis and Cirrhosis of Liver** | 1.09 (1.09, 1.10) | <0.001 | 1772.57 (1011.12, 3107.45) | <0.001 | 0.96 (0.96, 0.97) | <0.001 | 1.04 (1.04, 1.05) | <0.001 | 1.04 (0.99, 1.09) | 0.081 | 1.01 (1.01, 1.01) | <0.001 |
| **Hepatic Failure** | 1.06 (1.05, 1.07) | <0.001 | 249.93 (103.03, 606.27) | <0.001 | 0.97 (0.96, 0.98) | <0.001 | 1.03 (1.03, 1.04) | <0.001 | 1.17 (1.11, 1.24) | <0.001 | 1.01 (1.00, 1.01) | <0.001 |
| **GERD** | 1.03 (1.03, 1.04) | <0.001 | 14.87 (12.98, 17.02) | <0.001 | 0.99 (0.98, 0.99) | <0.001 | 1.02 (1.02, 1.02) | <0.001 | 1.04 (1.03, 1.05) | <0.001 | 1.00 (1.00, 1.00) | <0.001 |
| **IBS** | 1.02 (1.01, 1.02) | <0.001 | 4.24 (3.21, 5.62) | <0.001 | 0.97 (0.97, 0.98) | <0.001 | 1.01 (1.01, 1.02) | <0.001 | 1.03 (1.01, 1.06) | 0.005 | 1.00 (1.00, 1.00) | <0.001 |
| **Dyspepsia** | 1.00 (0.99, 1.00) | 0.201 | 1.17 (0.86, 1.58) | 0.322 | 0.99 (0.99, 0.99) | <0.001 | 1.00 (1.00, 1.01) | 0.329 | 1.01 (0.99, 1.03) | 0.502 | 1.00 (1.00, 1.00) | 0.616 |
| **Functional Intestinal Disorders** | 1.02 (1.02, 1.02) | <0.001 | 4.89 (4.09, 5.85) | <0.001 | 0.98 (0.98, 0.98) | <0.001 | 1.01 (1.01, 1.02) | <0.001 | 1.08 (1.07, 1.10) | <0.001 | 1.00 (1.00, 1.00) | <0.001 |
| **Oesophagitis** | 1.02 (1.01, 1.02) | <0.001 | 7.31 (5.49, 9.74) | <0.001 | 0.98 (0.98, 0.99) | <0.001 | 1.02 (1.01, 1.02) | <0.001 | 1.06 (1.04, 1.09) | <0.001 | 1.00 (1.00, 1.00) | <0.001 |
| **Other Oesophagus Diseases** | 1.01 (1.01, 1.02) | <0.001 | 5.95 (4.67, 7.57) | <0.001 | 0.99 (0.98, 0.99) | <0.001 | 1.01 (1.01, 1.02) | <0.001 | 1.08 (1.06, 1.10) | <0.001 | 1.00 (1.00, 1.00) | <0.001 |
| **Other Non Infective Gastroenteritis and Colitis** | 1.02 (1.02, 1.02) | <0.001 | 6.05 (4.77, 7.66) | <0.001 | 0.98 (0.98, 0.99) | <0.001 | 1.02 (1.02, 1.02) | <0.001 | 1.10 (1.09, 1.12) | <0.001 | 1.00 (1.00, 1.00) | <0.001 |
| **Anus and Rectum Diseases** | 1.02 (1.02, 1.02) | <0.001 | 4.59 (3.82, 5.52) | <0.001 | 0.99 (0.99, 1.00) | <0.001 | 1.01 (1.01, 1.01) | <0.001 | 1.05 (1.04, 1.07) | <0.001 | 1.00 (1.00, 1.00) | <0.001 |
| **Haemorrhoids** | 1.01 (1.01, 1.01) | <0.001 | 2.75 (2.25, 3.35) | <0.001 | 0.99 (0.99, 0.99) | <0.001 | 1.01 (1.00, 1.01) | 0.001 | 1.02 (1.00, 1.03) | 0.019 | 1.00 (1.00, 1.00) | <0.001 |
| **GI Diseases Excluding Cancer** | 1.02 (1.02, 1.02) | <0.001 | 5.73 (5.30, 6.20) | <0.001 | 0.99 (0.99, 0.99) | <0.001 | 1.01 (1.01, 1.02) | <0.001 | 1.05 (1.05, 1.06) | <0.001 | 1.00 (1.00, 1.00) | <0.001 |
| **GI Diseases** | 1.02 (1.02, 1.02) | <0.001 | 5.71 (5.28, 6.17) | <0.001 | 0.99 (0.99, 0.99) | <0.001 | 1.01 (1.01, 1.02) | <0.001 | 1.05 (1.05, 1.06) | <0.001 | 1.00 (1.00, 1.00) | <0.001 |

HR was calculated by Cox model adjusted for age, age squared, sex, ethnicity, Townsend deprivation index, smoking status, alcohol drinking, education level, physical activity, medicinal intake (aspirin, non-aspirin NSAIDs, and lipid-lowering drugs use), and comorbidities (lipidaemia, hypertension, diabetes).

**Table S2.** Subgroup analyses for the association between clusters and risk of digestive diseases stratified by age

| **Diseases** | **Age** | **Inflammatory Status** | | **Overweight with high strength** | | **Obesity with Insulin Resistant** | |
| --- | --- | --- | --- | --- | --- | --- | --- |
|  |  | **HR 95%CI** | ***P* value** | **HR 95%CI** | ***P* value** | **HR 95%CI** | ***P* value** |
| **Oesophagus Cancer** | <=45 | 0.00 (0.00, Inf) | 0.999 | 0.97 (0.29, 3.27) | 0.959 | 1.42 (0.38, 5.34) | 0.605 |
|  | >45 | 1.92 (1.37, 2.70) | <0.001 | 1.05 (0.87, 1.26) | 0.607 | 1.36 (1.13, 1.63) | 0.001 |
| **Stomach Cancer** | <=45 | 0.00 (0.00, Inf) | 0.998 | 1.83 (0.55, 6.05) | 0.322 | 1.55 (0.35, 6.80) | 0.560 |
|  | >45 | 1.71 (1.08, 2.71) | 0.022 | 1.30 (1.04, 1.63) | 0.023 | 1.57 (1.26, 1.95) | <0.001 |
| **Smallintestine Cancer** | <=45 | 0.00 (0.00, Inf) | 1.000 | 1.98 (0.21, 18.36) | 0.550 | 1.69 (0.14, 20.28) | 0.679 |
|  | >45 | 1.33 (0.61, 2.90) | 0.475 | 0.85 (0.59, 1.23) | 0.384 | 1.34 (0.97, 1.86) | 0.079 |
| **Colorectal Cancer** | <=45 | 0.28 (0.04, 1.99) | 0.202 | 0.70 (0.48, 1.02) | 0.066 | 1.07 (0.73, 1.58) | 0.720 |
|  | >45 | 1.49 (1.27, 1.75) | <0.001 | 1.04 (0.96, 1.12) | 0.352 | 1.17 (1.09, 1.26) | <0.001 |
| **Anus Cancer** | <=45 | 0.00 (0.00, Inf) | 0.999 | 1.18 (0.25, 5.53) | 0.833 | 0.89 (0.17, 4.59) | 0.887 |
|  | >45 | 1.66 (0.76, 3.63) | 0.200 | 0.85 (0.54, 1.33) | 0.477 | 0.88 (0.60, 1.30) | 0.528 |
| **Liver Cancer** | <=45 | 0.00 (0.00, Inf) | 0.999 | 0.26 (0.06, 1.19) | 0.083 | 0.51 (0.10, 2.59) | 0.415 |
|  | >45 | 1.78 (1.14, 2.77) | 0.011 | 0.63 (0.49, 0.81) | <0.001 | 1.73 (1.39, 2.14) | <0.001 |
| **Gallbladder Cancer** | <=45 | 0.00 (0.00, Inf) | 1.000 | 0.00 (0.00, Inf) | 0.999 | 1.95 (0.12, 31.14) | 0.637 |
|  | >45 | 1.10 (0.26, 4.59) | 0.897 | 1.54 (0.85, 2.79) | 0.157 | 2.27 (1.45, 3.56) | <0.001 |
| **Biliarytract Cancer** | <=45 | 0.00 (0.00, Inf) | 1.000 | 0.00 (0.00, Inf) | 0.999 | 1.59 (0.14, 17.59) | 0.703 |
|  | >45 | 1.20 (0.43, 3.33) | 0.732 | 1.13 (0.73, 1.75) | 0.594 | 1.39 (0.93, 2.08) | 0.105 |
| **Pancreas Cancer** | <=45 | 14.83 (3.47, 63.38) | <0.001 | 3.02 (0.92, 9.94) | 0.069 | 1.79 (0.47, 6.87) | 0.396 |
|  | >45 | 1.39 (0.96, 1.99) | 0.079 | 1.20 (1.02, 1.42) | 0.027 | 1.49 (1.28, 1.73) | <0.001 |
| **GI Cancer** | <=45 | 0.78 (0.29, 2.11) | 0.621 | 0.84 (0.62, 1.15) | 0.275 | 1.11 (0.81, 1.54) | 0.518 |
|  | >45 | 1.53 (1.35, 1.73) | <0.001 | 1.04 (0.98, 1.10) | 0.218 | 1.29 (1.23, 1.37) | <0.001 |
| **IBD** | <=45 | 1.71 (0.90, 3.26) | 0.102 | 1.10 (0.84, 1.44) | 0.494 | 1.07 (0.79, 1.45) | 0.644 |
|  | >45 | 2.63 (2.21, 3.12) | <0.001 | 1.07 (0.96, 1.19) | 0.212 | 1.12 (1.02, 1.23) | 0.022 |
| **Gastritis and Duodenitis** | <=45 | 1.43 (1.08, 1.90) | 0.012 | 1.10 (0.99, 1.23) | 0.082 | 1.54 (1.38, 1.71) | <0.001 |
|  | >45 | 1.31 (1.22, 1.41) | <0.001 | 1.00 (0.97, 1.03) | 0.973 | 1.23 (1.20, 1.27) | <0.001 |
| **Gastritis** | <=45 | 1.43 (1.05, 1.94) | 0.022 | 1.07 (0.94, 1.21) | 0.295 | 1.50 (1.34, 1.69) | <0.001 |
|  | >45 | 1.35 (1.25, 1.46) | <0.001 | 0.98 (0.94, 1.01) | 0.184 | 1.21 (1.18, 1.25) | <0.001 |
| **Duodenitis** | <=45 | 1.57 (0.93, 2.65) | 0.095 | 1.20 (0.98, 1.48) | 0.078 | 1.61 (1.30, 1.99) | <0.001 |
|  | >45 | 1.35 (1.17, 1.55) | <0.001 | 1.11 (1.04, 1.18) | 0.002 | 1.33 (1.25, 1.41) | <0.001 |
| **Acute Pancreatitis** | <=45 | 3.28 (1.48, 7.27) | 0.003 | 1.43 (0.94, 2.19) | 0.098 | 3.00 (2.05, 4.39) | <0.001 |
|  | >45 | 1.96 (1.53, 2.52) | <0.001 | 1.28 (1.13, 1.46) | <0.001 | 2.20 (1.98, 2.46) | <0.001 |
| **Appendicitis** | <=45 | 1.43 (0.70, 2.92) | 0.328 | 1.02 (0.78, 1.34) | 0.881 | 1.46 (1.11, 1.94) | 0.007 |
|  | >45 | 0.96 (0.70, 1.33) | 0.828 | 1.13 (1.00, 1.28) | 0.042 | 1.04 (0.93, 1.17) | 0.471 |
| **Cholecystitis** | <=45 | 4.14 (2.87, 5.96) | <0.001 | 2.05 (1.69, 2.49) | <0.001 | 4.27 (3.61, 5.05) | <0.001 |
|  | >45 | 2.46 (2.17, 2.79) | <0.001 | 1.60 (1.50, 1.72) | <0.001 | 2.48 (2.34, 2.62) | <0.001 |
| **Cholelithiasis** | <=45 | 4.31 (3.21, 5.78) | <0.001 | 1.95 (1.66, 2.29) | <0.001 | 4.20 (3.66, 4.81) | <0.001 |
|  | >45 | 2.37 (2.15, 2.62) | <0.001 | 1.55 (1.47, 1.64) | <0.001 | 2.40 (2.30, 2.51) | <0.001 |
| **Cholangitis** | <=45 | 4.61 (1.33, 16.02) | 0.016 | 1.67 (0.78, 3.57) | 0.189 | 2.25 (1.08, 4.66) | 0.029 |
|  | >45 | 2.65 (2.05, 3.43) | <0.001 | 1.44 (1.25, 1.67) | <0.001 | 1.94 (1.70, 2.22) | <0.001 |
| **ALD** | <=45 | 0.91 (0.22, 3.80) | 0.896 | 0.76 (0.47, 1.21) | 0.248 | 1.82 (1.13, 2.92) | 0.013 |
|  | >45 | 3.04 (2.29, 4.03) | <0.001 | 0.88 (0.73, 1.06) | 0.185 | 1.98 (1.66, 2.35) | <0.001 |
| **NAFLD** | <=45 | 5.80 (3.71, 9.09) | <0.001 | 2.04 (1.56, 2.66) | <0.001 | 6.68 (5.28, 8.44) | <0.001 |
|  | >45 | 4.02 (3.45, 4.69) | <0.001 | 1.69 (1.53, 1.85) | <0.001 | 4.76 (4.41, 5.14) | <0.001 |
| **Fibrosis and Cirrhosis of Liver** | <=45 | 4.86 (2.10, 11.25) | <0.001 | 0.91 (0.51, 1.61) | 0.740 | 2.53 (1.52, 4.21) | <0.001 |
|  | >45 | 2.86 (2.23, 3.65) | <0.001 | 0.80 (0.68, 0.94) | 0.006 | 2.64 (2.32, 3.00) | <0.001 |
| **Hepatic Failure** | <=45 | 1.29 (0.17, 9.95) | 0.808 | 0.83 (0.37, 1.88) | 0.656 | 0.95 (0.37, 2.44) | 0.916 |
|  | >45 | 2.85 (2.00, 4.05) | <0.001 | 1.06 (0.86, 1.32) | 0.580 | 2.04 (1.68, 2.48) | <0.001 |
| **GERD** | <=45 | 2.18 (1.73, 2.74) | <0.001 | 1.41 (1.27, 1.56) | <0.001 | 2.02 (1.83, 2.23) | <0.001 |
|  | >45 | 1.44 (1.34, 1.54) | <0.001 | 1.21 (1.17, 1.25) | <0.001 | 1.48 (1.44, 1.52) | <0.001 |
| **IBS** | <=45 | 1.89 (1.30, 2.76) | <0.001 | 0.90 (0.74, 1.08) | 0.265 | 1.49 (1.27, 1.75) | <0.001 |
|  | >45 | 1.17 (1.01, 1.36) | 0.042 | 0.93 (0.87, 1.00) | 0.045 | 1.19 (1.12, 1.25) | <0.001 |
| **Dyspepsia** | <=45 | 1.31 (0.77, 2.25) | 0.320 | 1.12 (0.91, 1.39) | 0.274 | 1.31 (1.06, 1.61) | 0.011 |
|  | >45 | 1.01 (0.86, 1.18) | 0.923 | 0.96 (0.90, 1.03) | 0.222 | 0.98 (0.92, 1.04) | 0.427 |
| **Functional Intestinal Disorders** | <=45 | 1.15 (0.78, 1.69) | 0.481 | 0.97 (0.84, 1.13) | 0.706 | 1.30 (1.13, 1.49) | <0.001 |
|  | >45 | 1.32 (1.22, 1.44) | <0.001 | 0.87 (0.84, 0.91) | <0.001 | 1.18 (1.14, 1.22) | <0.001 |
| **Oesophagitis** | <=45 | 2.73 (1.72, 4.35) | <0.001 | 1.42 (1.14, 1.78) | 0.002 | 1.97 (1.57, 2.47) | <0.001 |
|  | >45 | 1.40 (1.22, 1.61) | <0.001 | 1.13 (1.06, 1.21) | <0.001 | 1.27 (1.20, 1.34) | <0.001 |
| **Other Oesophagus Diseases** | <=45 | 1.59 (0.98, 2.57) | 0.058 | 1.26 (1.04, 1.53) | 0.016 | 1.41 (1.15, 1.72) | <0.001 |
|  | >45 | 1.60 (1.44, 1.78) | <0.001 | 1.15 (1.09, 1.21) | <0.001 | 1.23 (1.17, 1.29) | <0.001 |
| **Other Non Infective Gastroenteritis and Colitis** | <=45 | 1.87 (1.29, 2.72) | <0.001 | 1.07 (0.91, 1.27) | 0.399 | 1.51 (1.29, 1.78) | <0.001 |
|  | >45 | 1.58 (1.42, 1.76) | <0.001 | 0.96 (0.91, 1.01) | 0.122 | 1.22 (1.16, 1.28) | <0.001 |
| **Anus and Rectum Diseases** | <=45 | 1.13 (0.80, 1.60) | 0.475 | 1.24 (1.11, 1.39) | <0.001 | 1.40 (1.24, 1.58) | <0.001 |
|  | >45 | 1.32 (1.21, 1.45) | <0.001 | 1.04 (1.00, 1.08) | 0.054 | 1.23 (1.19, 1.28) | <0.001 |
| **Haemorrhoids** | <=45 | 1.09 (0.78, 1.53) | 0.621 | 1.04 (0.92, 1.17) | 0.558 | 1.10 (0.97, 1.25) | 0.133 |
|  | >45 | 1.07 (0.96, 1.19) | 0.209 | 0.99 (0.95, 1.04) | 0.698 | 1.17 (1.12, 1.21) | <0.001 |
| **GI Diseases Excluding Cancer** | <=45 | 1.48 (1.30, 1.70) | <0.001 | 1.20 (1.15, 1.26) | <0.001 | 1.54 (1.46, 1.62) | <0.001 |
|  | >45 | 1.39 (1.33, 1.44) | <0.001 | 1.07 (1.05, 1.09) | <0.001 | 1.26 (1.24, 1.28) | <0.001 |
| **GI Diseases** | <=45 | 1.47 (1.28, 1.68) | <0.001 | 1.21 (1.15, 1.27) | <0.001 | 1.53 (1.46, 1.61) | <0.001 |
|  | >45 | 1.38 (1.33, 1.44) | <0.001 | 1.07 (1.05, 1.09) | <0.001 | 1.26 (1.24, 1.28) | <0.001 |

HR was calculated by Cox model adjusted for age, age squared, sex, ethnicity, Townsend deprivation index, smoking status, alcohol drinking, education level, physical activity, medicinal intake (aspirin, non-aspirin NSAIDs, and lipid-lowering drugs use), and comorbidities (lipidaemia, hypertension, diabetes).

**Table S3.** Subgroup analyses for the association between clusters and risk of digestive diseases stratified by sex

| **Diseases** | **Sex** | **Inflammatory Status** | | **Overweight with high strength** | | **Obesity with Insulin Resistant** | |
| --- | --- | --- | --- | --- | --- | --- | --- |
|  |  | **HR 95%CI** | ***P* value** | **HR 95%CI** | ***P* value** | **HR 95%CI** | ***P* value** |
| **Oesophagus Cancer** | Male | 2.23 (1.46-3.41) | <0.001 | 1.25 (0.98-1.59) | 0.067 | 1.61 (1.23-2.11) | 0.001 |
|  | Female | 1.62 (0.84-3.12) | 0.147 | 0.82 (0.49-1.37) | 0.452 | 1.03 (0.77-1.40) | 0.823 |
| **Stomach Cancer** | Male | 2.05 (1.14-3.70) | 0.017 | 1.51 (1.12-2.05) | 0.008 | 1.91 (1.36-2.68) | <0.001 |
|  | Female | 1.32 (0.57-3.04) | 0.511 | 1.25 (0.76-2.05) | 0.384 | 1.26 (0.92-1.71) | 0.148 |
| **Smallintestine Cancer** | Male | 1.91 (0.75-4.87) | 0.175 | 0.84 (0.51-1.38) | 0.487 | 1.28 (0.73-2.24) | 0.384 |
|  | Female | 0.41 (0.06-2.99) | 0.38 | 1.09 (0.55-2.19) | 0.804 | 1.18 (0.78-1.77) | 0.437 |
| **Colorectal Cancer** | Male | 1.51 (1.21-1.89) | <0.001 | 1.10 (1.00-1.22) | 0.061 | 1.33 (1.18-1.50) | <0.001 |
|  | Female | 1.49 (1.18-1.87) | 0.001 | 1.05 (0.91-1.22) | 0.503 | 1.04 (0.94-1.14) | 0.432 |
| **Anus Cancer** | Male | 2.83 (0.89-9.00) | 0.078 | 0.95 (0.47-1.91) | 0.882 | 1.04 (0.45-2.40) | 0.93 |
|  | Female | 0.99 (0.30-3.22) | 0.983 | 1.00 (0.53-1.89) | 0.993 | 0.80 (0.50-1.28) | 0.349 |
| **Liver Cancer** | Male | 1.46 (0.79-2.70) | 0.222 | 0.73 (0.53-1.00) | 0.053 | 1.76 (1.27-2.45) | 0.001 |
|  | Female | 2.50 (1.32-4.71) | 0.005 | 0.81 (0.44-1.51) | 0.514 | 1.13 (0.80-1.61) | 0.481 |
| **Gallbladder Cancer** | Male | 0.00 (0.00-0.00) | <0.001 | 0.98 (0.35-2.72) | 0.962 | 1.74 (0.59-5.16) | 0.315 |
|  | Female | 1.69 (0.41-6.96) | 0.465 | 2.42 (1.14-5.11) | 0.021 | 2.34 (1.40-3.94) | 0.001 |
| **Biliarytract Cancer** | Male | 0.94 (0.22-4.09) | 0.934 | 0.98 (0.56-1.71) | 0.933 | 1.14 (0.59-2.20) | 0.706 |
|  | Female | 1.50 (0.35-6.35) | 0.585 | 1.22 (0.52-2.84) | 0.645 | 1.66 (1.03-2.68) | 0.039 |
| **Pancreas Cancer** | Male | 1.36 (0.79-2.33) | 0.267 | 1.25 (0.99-1.59) | 0.066 | 1.58 (1.20-2.08) | 0.001 |
|  | Female | 1.56 (0.97-2.48) | 0.065 | 1.34 (1.00-1.80) | 0.05 | 1.21 (0.99-1.48) | 0.059 |
| **GI Cancer** | Male | 1.60 (1.35-1.90) | <0.001 | 1.13 (1.04-1.22) | 0.003 | 1.46 (1.33-1.60) | <0.001 |
|  | Female | 1.49 (1.25-1.79) | <0.001 | 1.08 (0.96-1.21) | 0.189 | 1.11 (1.03-1.19) | 0.008 |
| **IBD** | Male | 1.29 (1.06-1.56) | 0.01 | 0.99 (0.91-1.08) | 0.866 | 0.89 (0.80-0.99) | 0.039 |
|  | Female | 1.33 (1.10-1.60) | 0.003 | 1.06 (0.95-1.19) | 0.284 | 1.33 (1.24-1.42) | <0.001 |
| **Gastritis and Duodenitis** | Male | 1.26 (1.13-1.41) | <0.001 | 1.08 (1.03-1.13) | 0.001 | 1.15 (1.09-1.21) | <0.001 |
|  | Female | 1.39 (1.28-1.52) | <0.001 | 1.16 (1.10-1.22) | <0.001 | 1.44 (1.40-1.49) | <0.001 |
| **Gastritis** | Male | 1.43 (1.23-1.66) | <0.001 | 1.02 (0.95-1.09) | 0.532 | 0.95 (0.87-1.03) | 0.215 |
|  | Female | 1.48 (1.27-1.72) | <0.001 | 1.13 (1.03-1.24) | 0.011 | 1.24 (1.16-1.32) | <0.001 |
| **Duodenitis** | Male | 1.13 (1.01-1.26) | 0.028 | 0.94 (0.89-0.98) | 0.006 | 1.00 (0.94-1.06) | 0.968 |
|  | Female | 1.26 (1.15-1.38) | <0.001 | 0.96 (0.90-1.01) | 0.113 | 1.12 (1.08-1.16) | <0.001 |
| **Acute Pancreatitis** | Male | 0.88 (0.68-1.15) | 0.349 | 0.91 (0.82-1.01) | 0.064 | 0.82 (0.73-0.93) | 0.002 |
|  | Female | 1.01 (0.84-1.22) | 0.915 | 0.94 (0.84-1.04) | 0.205 | 0.97 (0.90-1.03) | 0.32 |
| **Appendicitis** | Male | 1.39 (0.91-2.12) | 0.132 | 1.28 (1.08-1.52) | 0.005 | 1.31 (1.06-1.62) | 0.013 |
|  | Female | 0.84 (0.55-1.28) | 0.416 | 1.02 (0.85-1.23) | 0.843 | 1.05 (0.92-1.21) | 0.449 |
| **Cholecystitis** | Male | 2.39 (1.83-3.12) | <0.001 | 1.07 (0.93-1.24) | 0.359 | 1.12 (0.94-1.34) | 0.197 |
|  | Female | 2.53 (2.03-3.14) | <0.001 | 1.05 (0.89-1.25) | 0.561 | 1.02 (0.91-1.15) | 0.69 |
| **Cholelithiasis** | Male | 1.14 (1.01-1.29) | 0.034 | 0.91 (0.86-0.95) | <0.001 | 0.98 (0.92-1.04) | 0.465 |
|  | Female | 1.29 (1.18-1.43) | <0.001 | 0.95 (0.90-1.01) | 0.118 | 1.10 (1.06-1.14) | <0.001 |
| **Cholangitis** | Male | 1.13 (0.93-1.37) | 0.22 | 1.01 (0.93-1.09) | 0.859 | 1.03 (0.94-1.14) | 0.524 |
|  | Female | 1.33 (1.09-1.62) | 0.004 | 1.02 (0.90-1.15) | 0.783 | 1.25 (1.16-1.35) | <0.001 |
| **ALD** | Male | 1.40 (1.18-1.67) | <0.001 | 0.94 (0.87-1.02) | 0.118 | 1.04 (0.94-1.14) | 0.443 |
|  | Female | 1.55 (1.36-1.76) | <0.001 | 0.92 (0.85-1.01) | 0.081 | 1.12 (1.06-1.18) | <0.001 |
| **NAFLD** | Male | 0.92 (0.68-1.25) | 0.595 | 0.88 (0.79-0.99) | 0.033 | 0.98 (0.85-1.13) | 0.81 |
|  | Female | 1.19 (1.01-1.39) | 0.035 | 0.83 (0.75-0.91) | <0.001 | 1.11 (1.04-1.17) | 0.001 |
| **Fibrosis and Cirrhosis of Liver** | Male | 1.16 (1.03-1.32) | 0.018 | 0.85 (0.81-0.90) | <0.001 | 1.06 (1.00-1.13) | 0.07 |
|  | Female | 1.28 (1.15-1.42) | <0.001 | 0.90 (0.84-0.96) | 0.002 | 1.05 (1.00-1.09) | 0.03 |
| **Hepatic Failure** | Male | 1.31 (1.15-1.48) | <0.001 | 1.07 (1.02-1.13) | 0.008 | 1.22 (1.14-1.30) | <0.001 |
|  | Female | 1.24 (1.10-1.40) | 0.001 | 1.03 (0.96-1.11) | 0.356 | 1.12 (1.07-1.18) | <0.001 |
| **GERD** | Male | 0.98 (0.84-1.15) | 0.827 | 0.94 (0.89-1.00) | 0.036 | 1.01 (0.94-1.08) | 0.871 |
|  | Female | 1.04 (0.91-1.20) | 0.572 | 0.96 (0.89-1.03) | 0.285 | 1.12 (1.07-1.18) | <0.001 |
| **IBS** | Male | 2.05 (1.45-2.88) | <0.001 | 0.77 (0.63-0.94) | 0.011 | 1.40 (1.13-1.75) | 0.002 |
|  | Female | 3.72 (2.28-6.06) | <0.001 | 1.02 (0.64-1.63) | 0.942 | 1.68 (1.26-2.24) | <0.001 |
| **Dyspepsia** | Male | 2.33 (1.43-3.80) | 0.001 | 1.12 (0.85-1.48) | 0.433 | 1.74 (1.27-2.38) | <0.001 |
|  | Female | 2.84 (1.73-4.68) | <0.001 | 0.57 (0.31-1.05) | 0.073 | 1.38 (1.06-1.81) | 0.019 |
| **Functional Intestinal Disorders** | Male | 2.92 (2.25-3.79) | <0.001 | 1.55 (1.33-1.80) | <0.001 | 3.85 (3.28-4.52) | <0.001 |
|  | Female | 4.11 (3.43-4.92) | <0.001 | 1.89 (1.64-2.18) | <0.001 | 3.74 (3.41-4.10) | <0.001 |
| **Oesophagitis** | Male | 1.81 (1.25-2.62) | 0.002 | 0.75 (0.61-0.92) | 0.005 | 1.95 (1.58-2.41) | <0.001 |
|  | Female | 3.62 (2.63-4.97) | <0.001 | 0.86 (0.60-1.22) | 0.386 | 1.98 (1.66-2.36) | <0.001 |
| **Other Oesophagus Diseases** | Male | 2.44 (1.98-3.01) | <0.001 | 1.53 (1.36-1.71) | <0.001 | 2.17 (1.91-2.47) | <0.001 |
|  | Female | 2.43 (2.09-2.83) | <0.001 | 1.63 (1.48-1.80) | <0.001 | 2.59 (2.44-2.76) | <0.001 |
| **Other Non Infective Gastroenteritis and Colitis** | Male | 2.29 (1.93-2.72) | <0.001 | 1.51 (1.38-1.65) | <0.001 | 2.11 (1.91-2.34) | <0.001 |
|  | Female | 2.39 (2.13-2.68) | <0.001 | 1.55 (1.44-1.67) | <0.001 | 2.45 (2.34-2.57) | <0.001 |
| **Anus and Rectum Diseases** | Male | 2.25 (1.50-3.37) | <0.001 | 1.43 (1.15-1.78) | 0.001 | 1.81 (1.42-2.32) | <0.001 |
|  | Female | 2.71 (1.94-3.78) | <0.001 | 1.39 (1.07-1.81) | 0.014 | 1.57 (1.33-1.86) | <0.001 |
| **Haemorrhoids** | Male | 1.70 (1.17-2.48) | 0.006 | 1.19 (0.99-1.44) | 0.067 | 1.92 (1.56-2.37) | <0.001 |
|  | Female | 2.06 (1.49-2.84) | <0.001 | 1.38 (1.11-1.70) | 0.003 | 2.11 (1.86-2.41) | <0.001 |
| **GI Diseases Excluding Cancer** | Male | 1.27 (1.20-1.35) | <0.001 | 1.01 (0.98-1.03) | 0.515 | 1.08 (1.05-1.11) | <0.001 |
|  | Female | 1.34 (1.27-1.42) | <0.001 | 1.07 (1.04-1.10) | <0.001 | 1.23 (1.21-1.25) | <0.001 |
| **GI Diseases** | Male | 1.27 (1.20-1.35) | <0.001 | 1.01 (0.99-1.03) | 0.486 | 1.08 (1.05-1.12) | <0.001 |
|  | Female | 1.34 (1.27-1.41) | <0.001 | 1.07 (1.04-1.10) | <0.001 | 1.23 (1.20-1.25) | <0.001 |

HR was calculated by Cox model adjusted for age, age squared, sex, ethnicity, Townsend deprivation index, smoking status, alcohol drinking, education level, physical activity, medicinal intake (aspirin, non-aspirin NSAIDs, and lipid-lowering drugs use), and comorbidities (lipidaemia, hypertension, diabetes).

**Table S4.** Subgroup analyses for the association between clusters and risk of digestive diseases stratified by ethnicity

| **Diseases** | **Ethnicity** | **Inflammatory Status** | | **Overweight with high strength** | | **Obesity with Insulin Resistant** | |
| --- | --- | --- | --- | --- | --- | --- | --- |
|  |  | **HR 95%CI** | ***P* value** | **HR 95%CI** | ***P* value** | **HR 95%CI** | ***P* value** |
| **Oesophagus Cancer** | White | 1.78 (1.25, 2.53) | 0.001 | 1.03 (0.86, 1.24) | 0.763 | 1.39 (1.16, 1.67) | <0.001 |
|  | Other | 19.09 (3.80, 96.03) | <0.001 | 3.40 (0.80, 14.43) | 0.097 | 0.92 (0.15, 5.57) | 0.929 |
| **Stomach Cancer** | White | 1.61 (1.01, 2.58) | 0.046 | 1.25 (0.99, 1.57) | 0.058 | 1.49 (1.20, 1.86) | <0.001 |
|  | Other | 4.34 (0.50, 37.39) | 0.181 | 3.49 (1.15, 10.59) | 0.027 | 4.23 (1.51, 11.85) | 0.006 |
| **Smallintestine Cancer** | White | 1.19 (0.51, 2.74) | 0.686 | 0.84 (0.58, 1.23) | 0.378 | 1.39 (1.00, 1.93) | 0.049 |
|  | Other | 5.67 (0.58, 55.11) | 0.135 | 1.62 (0.33, 7.94) | 0.553 | 1.21 (0.24, 6.09) | 0.814 |
| **Colorectal Cancer** | White | 1.48 (1.26, 1.74) | <0.001 | 1.03 (0.95, 1.11) | 0.490 | 1.18 (1.10, 1.27) | <0.001 |
|  | Other | 0.78 (0.25, 2.47) | 0.672 | 0.86 (0.58, 1.27) | 0.450 | 0.98 (0.69, 1.39) | 0.911 |
| **Anus Cancer** | White | 1.34 (0.59, 3.09) | 0.485 | 0.87 (0.56, 1.34) | 0.522 | 0.88 (0.61, 1.28) | 0.507 |
|  | Other | 2.18e+09 (1.36e+08, 3.48e+10) | <0.001 | 5.78e+07 (3.62e+06, 9.25 e+08) | <0.001 | 0.85 (0.00, Inf) | 1.000 |
| **Liver Cancer** | White | 1.88 (1.20, 2.93) | 0.006 | 0.64 (0.50, 0.83) | <0.001 | 1.81 (1.46, 2.25) | <0.001 |
|  | Other | 0.00 (0.00, Inf) | 0.997 | 0.35 (0.13, 0.99) | 0.047 | 0.67 (0.25, 1.79) | 0.419 |
| **Gallbladder Cancer** | White | 1.15 (0.27, 4.78) | 0.852 | 1.53 (0.84, 2.78) | 0.169 | 2.31 (1.47, 3.63) | <0.001 |
|  | Other | 0.00 (0.00, Inf) | 1.000 | 1.51 (0.07, 30.37) | 0.789 | 4.03 (0.41, 39.72) | 0.233 |
| **Biliarytract Cancer** | White | 0.93 (0.29, 2.99) | 0.898 | 1.14 (0.73, 1.79) | 0.565 | 1.43 (0.95, 2.14) | 0.086 |
|  | Other | 6.73 (0.68, 66.17) | 0.102 | 0.58 (0.09, 3.85) | 0.571 | 1.47 (0.29, 7.49) | 0.641 |
| **Pancreas Cancer** | White | 1.53 (1.07, 2.17) | 0.019 | 1.21 (1.02, 1.43) | 0.029 | 1.54 (1.32, 1.79) | <0.001 |
|  | Other | 1.12 (0.15, 8.36) | 0.915 | 1.90 (0.94, 3.87) | 0.075 | 0.97 (0.48, 1.98) | 0.939 |
| **GI Cancer** | White | 1.51 (1.33, 1.70) | <0.001 | 1.03 (0.97, 1.09) | 0.370 | 1.31 (1.24, 1.38) | <0.001 |
|  | Other | 1.84 (0.99, 3.41) | 0.054 | 1.12 (0.84, 1.49) | 0.449 | 1.12 (0.86, 1.47) | 0.408 |
| **IBD** | White | 2.56 (2.15, 3.03) | <0.001 | 1.09 (0.99, 1.21) | 0.087 | 1.16 (1.05, 1.27) | 0.002 |
|  | Other | 3.01 (1.55, 5.87) | 0.001 | 0.90 (0.61, 1.34) | 0.613 | 0.76 (0.50, 1.14) | 0.185 |
| **Gastritis and Duodenitis** | White | 1.35 (1.26, 1.45) | <0.001 | 1.02 (0.98, 1.05) | 0.329 | 1.28 (1.24, 1.31) | <0.001 |
|  | Other | 1.18 (0.87, 1.59) | 0.281 | 0.93 (0.82, 1.05) | 0.222 | 1.17 (1.05, 1.30) | 0.004 |
| **Gastritis** | White | 1.38 (1.28, 1.49) | <0.001 | 0.99 (0.95, 1.03) | 0.577 | 1.25 (1.21, 1.29) | <0.001 |
|  | Other | 1.26 (0.92, 1.72) | 0.145 | 0.92 (0.81, 1.06) | 0.259 | 1.18 (1.05, 1.32) | 0.004 |
| **Duodenitis** | White | 1.39 (1.21, 1.60) | <0.001 | 1.13 (1.07, 1.21) | <0.001 | 1.37 (1.30, 1.46) | <0.001 |
|  | Other | 1.18 (0.64, 2.16) | 0.603 | 0.92 (0.73, 1.16) | 0.463 | 1.28 (1.03, 1.59) | 0.024 |
| **Acute Pancreatitis** | White | 2.19 (1.72, 2.79) | <0.001 | 1.32 (1.16, 1.50) | <0.001 | 2.36 (2.12, 2.62) | <0.001 |
|  | Other | 0.49 (0.07, 3.56) | 0.480 | 0.80 (0.44, 1.46) | 0.476 | 1.61 (1.05, 2.47) | 0.030 |
| **Appendicitis** | White | 1.08 (0.80, 1.45) | 0.617 | 1.14 (1.02, 1.28) | 0.023 | 1.11 (0.99, 1.23) | 0.069 |
|  | Other | 0.00 (0.00, Inf) | 0.994 | 0.64 (0.38, 1.09) | 0.102 | 0.86 (0.53, 1.39) | 0.541 |
| **Cholecystitis** | White | 2.68 (2.37, 3.03) | <0.001 | 1.69 (1.58, 1.80) | <0.001 | 2.71 (2.57, 2.86) | <0.001 |
|  | Other | 2.69 (1.57, 4.61) | <0.001 | 1.02 (0.75, 1.39) | 0.887 | 2.28 (1.80, 2.88) | <0.001 |
| **Cholelithiasis** | White | 2.61 (2.37, 2.87) | <0.001 | 1.61 (1.53, 1.70) | <0.001 | 2.61 (2.50, 2.72) | <0.001 |
|  | Other | 2.22 (1.38, 3.57) | <0.001 | 1.22 (0.95, 1.57) | 0.114 | 2.30 (1.90, 2.78) | <0.001 |
| **Cholangitis** | White | 2.73 (2.11, 3.54) | <0.001 | 1.48 (1.27, 1.72) | <0.001 | 2.05 (1.80, 2.34) | <0.001 |
|  | Other | 3.26 (1.13, 9.38) | 0.029 | 1.19 (0.64, 2.20) | 0.584 | 1.09 (0.60, 1.98) | 0.778 |
| **ALD** | White | 2.88 (2.18, 3.80) | <0.001 | 0.87 (0.73, 1.03) | 0.106 | 2.00 (1.69, 2.36) | <0.001 |
|  | Other | 3.21 (0.70, 14.74) | 0.133 | 0.80 (0.32, 2.00) | 0.637 | 2.52 (1.15, 5.49) | 0.020 |
| **NAFLD** | White | 4.19 (3.59, 4.88) | <0.001 | 1.76 (1.61, 1.93) | <0.001 | 5.24 (4.85, 5.65) | <0.001 |
|  | Other | 5.27 (3.32, 8.36) | <0.001 | 1.31 (0.94, 1.81) | 0.108 | 3.20 (2.48, 4.14) | <0.001 |
| **Fibrosis and Cirrhosis of Liver** | White | 3.06 (2.39, 3.91) | <0.001 | 0.83 (0.70, 0.97) | 0.018 | 2.86 (2.52, 3.26) | <0.001 |
|  | Other | 3.51 (1.45, 8.47) | 0.005 | 0.64 (0.35, 1.16) | 0.142 | 1.42 (0.85, 2.35) | 0.1800 |
| **Hepatic Failure** | White | 2.77 (1.93, 3.97) | <0.001 | 1.02 (0.82, 1.27) | 0.831 | 2.09 (1.72, 2.54) | <0.001 |
|  | Other | 4.01 (1.15, 14.05) | 0.030 | 1.48 (0.70, 3.13) | 0.302 | 1.66 (0.81, 3.40) | 0.163 |
| **GERD** | White | 1.51 (1.41, 1.62) | <0.001 | 1.24 (1.20, 1.28) | <0.001 | 1.55 (1.51, 1.59) | <0.001 |
|  | Other | 1.37 (1.02, 1.85) | 0.038 | 1.04 (0.91, 1.18) | 0.560 | 1.35 (1.22, 1.51) | <0.001 |
| **IBS** | White | 1.27 (1.10, 1.46) | 0.001 | 0.94 (0.88, 1.01) | 0.079 | 1.26 (1.20, 1.33) | <0.001 |
|  | Other | 1.28 (0.67, 2.42) | 0.453 | 0.77 (0.57, 1.05) | 0.103 | 1.03 (0.81, 1.31) | 0.833 |
| **Dyspepsia** | White | 1.04 (0.89, 1.21) | 0.650 | 0.98 (0.91, 1.04) | 0.483 | 1.00 (0.95, 1.07) | 0.869 |
|  | Other | 0.99 (0.55, 1.76) | 0.961 | 0.94 (0.74, 1.19) | 0.589 | 0.98 (0.81, 1.20) | 0.857 |
| **Functional Intestinal Disorders** | White | 1.34 (1.23, 1.45) | <0.001 | 0.88 (0.84, 0.91) | <0.001 | 1.20 (1.16, 1.24) | <0.001 |
|  | Other | 1.42 (1.02, 2.00) | 0.041 | 0.89 (0.76, 1.04) | 0.149 | 1.27 (1.12, 1.44) | <0.001 |
| **Oesophagitis** | White | 1.50 (1.31, 1.71) | <0.001 | 1.17 (1.10, 1.24) | <0.001 | 1.32 (1.25, 1.40) | <0.001 |
|  | Other | 1.25 (0.66, 2.38) | 0.487 | 0.94 (0.73, 1.23) | 0.673 | 1.37 (1.09, 1.71) | 0.007 |
| **Other Oesophagus Diseases** | White | 1.64 (1.48, 1.83) | <0.001 | 1.17 (1.11, 1.23) | <0.001 | 1.27 (1.21, 1.33) | <0.001 |
|  | Other | 1.09 (0.61, 1.95) | 0.775 | 0.94 (0.75, 1.18) | 0.600 | 1.06 (0.86, 1.30) | 0.608 |
| **Other Non Infective Gastroenteritis and Colitis** | White | 1.61 (1.45, 1.79) | <0.001 | 0.98 (0.93, 1.03) | 0.438 | 1.27 (1.22, 1.33) | <0.001 |
|  | Other | 2.14 (1.41, 3.23) | <0.001 | 0.87 (0.70, 1.09) | 0.238 | 1.16 (0.96, 1.40) | 0.136 |
| **Anus and Rectum Diseases** | White | 1.30 (1.19, 1.42) | <0.001 | 1.05 (1.01, 1.09) | 0.010 | 1.26 (1.21, 1.30) | <0.001 |
|  | Other | 1.72 (1.22, 2.43) | 0.002 | 1.20 (1.03, 1.40) | 0.019 | 1.22 (1.06, 1.41) | 0.006 |
| **Haemorrhoids** | White | 1.10 (0.99, 1.22) | 0.078 | 1.00 (0.96, 1.05) | 0.938 | 1.17 (1.13, 1.22) | <0.001 |
|  | Other | 0.81 (0.52, 1.27) | 0.366 | 0.96 (0.82, 1.11) | 0.564 | 1.11 (0.97, 1.27) | 0.128 |
| **GI Diseases Excluding Cancer** | White | 1.41 (1.35, 1.47) | <0.001 | 1.09 (1.07, 1.10) | <0.001 | 1.30 (1.28, 1.32) | <0.001 |
|  | Other | 1.49 (1.27, 1.75) | <0.001 | 1.07 (1.00, 1.14) | 0.053 | 1.28 (1.21, 1.36) | <0.001 |
| **GI Diseases** | White | 1.40 (1.35, 1.46) | <0.001 | 1.08 (1.07, 1.10) | <0.001 | 1.29 (1.28, 1.31) | <0.001 |
|  | Other | 1.50 (1.27, 1.76) | <0.001 | 1.07 (1.00, 1.14) | 0.056 | 1.28 (1.20, 1.36) | <0.001 |

HR was calculated by Cox model adjusted for age, age squared, sex, ethnicity, Townsend deprivation index, smoking status, alcohol drinking, education level, physical activity, medicinal intake (aspirin, non-aspirin NSAIDs, and lipid-lowering drugs use), and comorbidities (lipidaemia, hypertension, diabetes).

**Table S5.** Subgroup analyses for the association between clusters and risk of digestive diseases stratified by PRS for obesity

| **Diseases** | **PRS** | **Inflammatory Status** | | **Overweight with high strength** | | **Obesity with Insulin Resistant** | |
| --- | --- | --- | --- | --- | --- | --- | --- |
|  |  | **HR 95%CI** | ***P* value** | **HR 95%CI** | ***P* value** | **HR 95%CI** | ***P* value** |
| **Oesophagus Cancer** | High | 2.12 (1.30, 3.47) | 0.003 | 1.21 (0.94, 1.57) | 0.146 | 1.45 (1.09, 1.93) | 0.012 |
|  | Low | 1.58 (0.98, 2.54) | 0.058 | 0.87 (0.67, 1.13) | 0.305 | 1.12 (0.87, 1.44) | 0.375 |
| **Stomach Cancer** | High | 2.40 (1.30, 4.44) | 0.005 | 1.42 (1.03, 1.96) | 0.031 | 1.72 (1.22, 2.42) | 0.002 |
|  | Low | 1.13 (0.56, 2.28) | 0.724 | 1.18 (0.86, 1.62) | 0.298 | 1.32 (0.97, 1.78) | 0.074 |
| **Smallintestine Cancer** | High | 1.44 (0.52, 4.03) | 0.484 | 0.88 (0.52, 1.47) | 0.624 | 1.17 (0.72, 1.90) | 0.520 |
|  | Low | 1.10 (0.33, 3.66) | 0.879 | 0.85 (0.49, 1.48) | 0.574 | 1.33 (0.80, 2.19) | 0.271 |
| **Colorectal Cancer** | High | 1.57 (1.26, 1.96) | <0.001 | 1.06 (0.96, 1.18) | 0.250 | 1.16 (1.04, 1.29) | 0.010 |
|  | Low | 1.34 (1.07, 1.69) | 0.012 | 0.98 (0.88, 1.09) | 0.699 | 1.13 (1.02, 1.25) | 0.022 |
| **Anus Cancer** | High | 0.76 (0.18, 3.16) | 0.707 | 0.65 (0.35, 1.21) | 0.179 | 0.68 (0.38, 1.24) | 0.211 |
|  | Low | 2.74 (1.06, 7.11) | 0.038 | 1.20 (0.65, 2.22) | 0.558 | 1.10 (0.64, 1.89) | 0.725 |
| **Liver Cancer** | High | 1.07 (0.49, 2.33) | 0.862 | 0.61 (0.43, 0.87) | 0.006 | 1.40 (1.01, 1.96) | 0.046 |
|  | Low | 2.03 (1.16, 3.54) | 0.013 | 0.64 (0.45, 0.91) | 0.014 | 1.40 (1.02, 1.92) | 0.040 |
| **Gallbladder Cancer** | High | 1.19 (0.16, 8.94) | 0.867 | 1.80 (0.78, 4.15) | 0.165 | 2.61 (1.35, 5.04) | 0.004 |
|  | Low | 0.96 (0.13, 7.31) | 0.969 | 1.29 (0.56, 3.01) | 0.549 | 2.00 (1.04, 3.83) | 0.037 |
| **Biliarytract Cancer** | High | 0.65 (0.09, 4.84) | 0.678 | 1.22 (0.66, 2.24) | 0.524 | 1.26 (0.66, 2.39) | 0.483 |
|  | Low | 1.63 (0.48, 5.51) | 0.432 | 0.97 (0.51, 1.84) | 0.919 | 1.49 (0.85, 2.61) | 0.168 |
| **Pancreas Cancer** | High | 1.80 (1.13, 2.86) | 0.013 | 1.21 (0.96, 1.52) | 0.113 | 1.19 (0.93, 1.51) | 0.162 |
|  | Low | 1.14 (0.67, 1.93) | 0.641 | 1.20 (0.95, 1.52) | 0.124 | 1.44 (1.17, 1.78) | <0.001 |
| **GI Cancer** | High | 1.58 (1.33, 1.87) | <0.001 | 1.07 (0.99, 1.16) | 0.100 | 1.23 (1.13, 1.34) | <0.001 |
|  | Low | 1.40 (1.18, 1.66) | <0.001 | 0.99 (0.91, 1.07) | 0.745 | 1.22 (1.12, 1.31) | <0.001 |
| **IBD** | High | 2.42 (1.90, 3.09) | <0.001 | 1.00 (0.87, 1.14) | 0.958 | 1.02 (0.88, 1.18) | 0.808 |
|  | Low | 2.54 (2.02, 3.20) | <0.001 | 1.13 (0.98, 1.30) | 0.086 | 1.11 (0.97, 1.27) | 0.114 |
| **Gastritis and Duodenitis** | High | 1.23 (1.11, 1.36) | <0.001 | 0.98 (0.94, 1.03) | 0.512 | 1.07 (1.02, 1.12) | 0.003 |
|  | Low | 1.22 (1.11, 1.34) | <0.001 | 0.97 (0.93, 1.02) | 0.217 | 1.10 (1.05, 1.14) | <0.001 |
| **Gastritis** | High | 1.26 (1.13, 1.40) | <0.001 | 0.97 (0.93, 1.02) | 0.282 | 1.05 (1.00, 1.11) | 0.038 |
|  | Low | 1.24 (1.12, 1.37) | <0.001 | 0.93 (0.89, 0.98) | 0.008 | 1.07 (1.03, 1.12) | 0.002 |
| **Duodenitis** | High | 1.14 (0.93, 1.40) | 0.212 | 1.05 (0.97, 1.15) | 0.232 | 1.12 (1.02, 1.23) | 0.013 |
|  | Low | 1.37 (1.14, 1.64) | <0.001 | 1.10 (1.01, 1.20) | 0.030 | 1.21 (1.12, 1.31) | <0.001 |
| **Acute Pancreatitis** | High | 2.10 (1.49, 2.96) | <0.001 | 1.28 (1.07, 1.53) | 0.007 | 2.03 (1.73, 2.39) | <0.001 |
|  | Low | 1.75 (1.25, 2.45) | 0.001 | 1.26 (1.05, 1.50) | 0.011 | 2.02 (1.74, 2.35) | <0.001 |
| **Appendicitis** | High | 0.95 (0.61, 1.49) | 0.817 | 1.15 (0.99, 1.35) | 0.072 | 1.26 (1.07, 1.48) | 0.006 |
|  | Low | 1.08 (0.73, 1.59) | 0.714 | 1.07 (0.91, 1.25) | 0.437 | 1.01 (0.87, 1.18) | 0.887 |
| **Cholecystitis** | High | 2.37 (1.98, 2.84) | <0.001 | 1.60 (1.46, 1.76) | <0.001 | 2.39 (2.20, 2.59) | <0.001 |
|  | Low | 2.56 (2.18, 3.01) | <0.001 | 1.68 (1.54, 1.84) | <0.001 | 2.50 (2.32, 2.69) | <0.001 |
| **Cholelithiasis** | High | 2.26 (1.96, 2.60) | <0.001 | 1.59 (1.48, 1.71) | <0.001 | 2.27 (2.13, 2.42) | <0.001 |
|  | Low | 2.48 (2.19, 2.82) | <0.001 | 1.58 (1.48, 1.70) | <0.001 | 2.39 (2.25, 2.53) | <0.001 |
| **Cholangitis** | High | 2.81 (1.94, 4.05) | <0.001 | 1.58 (1.28, 1.94) | <0.001 | 1.65 (1.34, 2.04) | <0.001 |
|  | Low | 2.21 (1.56, 3.14) | <0.001 | 1.24 (1.01, 1.53) | 0.038 | 1.60 (1.34, 1.92) | <0.001 |
| **ALD** | High | 2.59 (1.76, 3.82) | <0.001 | 0.81 (0.64, 1.02) | 0.076 | 1.63 (1.28, 2.07) | <0.001 |
|  | Low | 2.40 (1.61, 3.57) | <0.001 | 0.85 (0.65, 1.10) | 0.218 | 1.52 (1.19, 1.96) | <0.001 |
| **NAFLD** | High | 3.40 (2.73, 4.23) | <0.001 | 1.56 (1.38, 1.78) | <0.001 | 3.42 (3.06, 3.82) | <0.001 |
|  | Low | 3.98 (3.26, 4.85) | <0.001 | 1.79 (1.58, 2.04) | <0.001 | 4.23 (3.80, 4.72) | <0.001 |
| **Fibrosis and Cirrhosis of Liver** | High | 2.27 (1.59, 3.25) | <0.001 | 0.75 (0.61, 0.93) | 0.010 | 1.74 (1.44, 2.12) | <0.001 |
|  | Low | 2.97 (2.15, 4.10) | <0.001 | 0.85 (0.68, 1.07) | 0.158 | 2.26 (1.87, 2.73) | <0.001 |
| **Hepatic Failure** | High | 3.02 (1.86, 4.89) | <0.001 | 1.08 (0.80, 1.45) | 0.632 | 1.40 (1.02, 1.91) | 0.036 |
|  | Low | 1.95 (1.18, 3.22) | 0.009 | 0.89 (0.66, 1.20) | 0.452 | 1.54 (1.18, 2.02) | 0.002 |
| **GERD** | High | 1.45 (1.31, 1.59) | <0.001 | 1.20 (1.15, 1.25) | <0.001 | 1.42 (1.37, 1.48) | <0.001 |
|  | Low | 1.32 (1.20, 1.45) | <0.001 | 1.17 (1.12, 1.22) | <0.001 | 1.32 (1.27, 1.37) | <0.001 |
| **IBS** | High | 1.21 (0.99, 1.48) | 0.067 | 0.90 (0.82, 0.99) | 0.030 | 1.10 (1.01, 1.19) | 0.026 |
|  | Low | 1.07 (0.88, 1.30) | 0.504 | 0.90 (0.82, 0.99) | 0.029 | 1.08 (1.01, 1.17) | 0.032 |
| **Dyspepsia** | High | 0.93 (0.75, 1.17) | 0.560 | 0.97 (0.88, 1.06) | 0.473 | 0.95 (0.87, 1.03) | 0.223 |
|  | Low | 1.01 (0.82, 1.24) | 0.930 | 0.94 (0.86, 1.04) | 0.230 | 0.93 (0.86, 1.00) | 0.064 |
| **Functional Intestinal Disorders** | High | 1.20 (1.07, 1.35) | 0.002 | 0.83 (0.79, 0.88) | <0.001 | 1.01 (0.96, 1.07) | 0.620 |
|  | Low | 1.24 (1.11, 1.39) | <0.001 | 0.87 (0.83, 0.92) | <0.001 | 1.06 (1.01, 1.11) | 0.014 |
| **Oesophagitis** | High | 1.42 (1.18, 1.72) | <0.001 | 1.15 (1.05, 1.25) | 0.002 | 1.18 (1.08, 1.29) | <0.001 |
|  | Low | 1.33 (1.11, 1.59) | 0.002 | 1.08 (0.99, 1.18) | 0.080 | 1.16 (1.07, 1.26) | <0.001 |
| **Other Oesophagus Diseases** | High | 1.54 (1.32, 1.79) | <0.001 | 1.18 (1.10, 1.27) | <0.001 | 1.17 (1.08, 1.26) | <0.001 |
|  | Low | 1.49 (1.29, 1.71) | <0.001 | 1.06 (0.99, 1.14) | 0.097 | 1.09 (1.02, 1.17) | 0.010 |
| **Other Non Infective Gastroenteritis and Colitis** | High | 1.46 (1.25, 1.70) | <0.001 | 0.95 (0.88, 1.02) | 0.177 | 1.04 (0.97, 1.12) | 0.245 |
|  | Low | 1.53 (1.33, 1.76) | <0.001 | 0.95 (0.88, 1.02) | 0.177 | 1.12 (1.05, 1.19) | <0.001 |
| **Anus and Rectum Diseases** | High | 1.19 (1.04, 1.35) | 0.011 | 1.05 (1.00, 1.11) | 0.049 | 1.19 (1.12, 1.25) | <0.001 |
|  | Low | 1.32 (1.17, 1.48) | <0.001 | 1.02 (0.97, 1.08) | 0.441 | 1.13 (1.07, 1.19) | <0.001 |
| **Haemorrhoids** | High | 1.02 (0.88, 1.19) | 0.776 | 0.93 (0.88, 0.99) | 0.018 | 1.05 (0.99, 1.12) | 0.101 |
|  | Low | 1.04 (0.90, 1.20) | 0.582 | 1.02 (0.96, 1.08) | 0.499 | 1.12 (1.06, 1.18) | <0.001 |
| **GI Diseases Excluding Cancer** | High | 1.33 (1.26, 1.41) | <0.001 | 1.05 (1.03, 1.08) | <0.001 | 1.17 (1.14, 1.20) | <0.001 |
|  | Low | 1.35 (1.28, 1.42) | <0.001 | 1.07 (1.05, 1.10) | <0.001 | 1.20 (1.18, 1.23) | <0.001 |
| **GI Diseases** | High | 1.33 (1.25, 1.40) | <0.001 | 1.05 (1.03, 1.08) | <0.001 | 1.17 (1.14, 1.20) | <0.001 |
|  | Low | 1.35 (1.28, 1.42) | <0.001 | 1.07 (1.05, 1.10) | <0.001 | 1.20 (1.18, 1.23) | <0.001 |

HR was calculated by Cox model adjusted for age, age squared, sex, ethnicity, Townsend deprivation index, smoking status, alcohol drinking, education level, physical activity, medicinal intake (aspirin, non-aspirin NSAIDs, and lipid-lowering drugs use), and comorbidities (lipidaemia, hypertension, diabetes).

**Table S6.** Sensitivity analyses for the association between clusters and risk of digestive diseases when further adjusted for family history of cancer

| **Diseases** | **Inflammatory Status** | | **Overweight with high strength** | | **Obesity with Insulin Resistant** | |
| --- | --- | --- | --- | --- | --- | --- |
|  | **HR 95%CI** | ***P* value** | **HR 95%CI** | ***P* value** | **HR 95%CI** | ***P* value** |
| **Oesophagus Cancer** | 1.86 (1.32-2.61) | <0.001 | 1.05 (0.88-1.25) | 0.608 | 1.29 (1.07-1.55) | 0.007 |
| **Stomach Cancer** | 1.68 (1.05-2.66) | 0.029 | 1.31 (1.05-1.63) | 0.015 | 1.53 (1.23-1.90) | <0.001 |
| **Smallintestine Cancer** | 1.24 (0.57-2.69) | 0.591 | 0.84 (0.57-1.21) | 0.34 | 1.22 (0.87-1.68) | 0.254 |
| **Colorectal Cancer** | 1.45 (1.24-1.70) | <0.001 | 1.02 (0.95-1.10) | 0.623 | 1.14 (1.06-1.23) | <0.001 |
| **Anus Cancer** | 1.55 (0.72-3.36) | 0.262 | 0.88 (0.57-1.37) | 0.578 | 0.84 (0.57-1.25) | 0.402 |
| **Liver Cancer** | 1.60 (1.03-2.49) | 0.038 | 0.62 (0.49-0.79) | <0.001 | 1.40 (1.12-1.74) | 0.003 |
| **Gallbladder Cancer** | 1.07 (0.26-4.46) | 0.926 | 1.52 (0.79-2.94) | 0.214 | 2.24 (1.40-3.57) | 0.001 |
| **Biliarytract Cancer** | 1.19 (0.43-3.33) | 0.741 | 1.08 (0.69-1.69) | 0.732 | 1.41 (0.94-2.09) | 0.095 |
| **Pancreas Cancer** | 1.44 (1.01-2.05) | 0.042 | 1.21 (1.02-1.42) | 0.027 | 1.35 (1.16-1.58) | <0.001 |
| **GI Cancer** | 1.49 (1.32-1.68) | <0.001 | 1.03 (0.97-1.09) | 0.365 | 1.23 (1.16-1.30) | <0.001 |
| **IBD** | 2.47 (2.09-2.92) | <0.001 | 1.06 (0.96-1.17) | 0.242 | 1.07 (0.97-1.17) | 0.202 |
| **Gastritis and Duodenitis** | 1.22 (1.14-1.31) | <0.001 | 0.98 (0.95-1.01) | 0.144 | 1.09 (1.05-1.12) | <0.001 |
| **Gastritis** | 1.25 (1.16-1.35) | <0.001 | 0.95 (0.92-0.99) | 0.007 | 1.07 (1.03-1.10) | <0.001 |
| **Duodenitis** | 1.26 (1.10-1.44) | 0.001 | 1.08 (1.01-1.15) | 0.017 | 1.17 (1.10-1.24) | <0.001 |
| **Acute Pancreatitis** | 1.92 (1.51-2.44) | <0.001 | 1.27 (1.12-1.44) | <0.001 | 2.07 (1.85-2.31) | <0.001 |
| **Appendicitis** | 1.02 (0.76-1.37) | 0.903 | 1.11 (0.99-1.24) | 0.064 | 1.10 (0.99-1.23) | 0.084 |
| **Cholecystitis** | 2.50 (2.21-2.81) | <0.001 | 1.65 (1.55-1.76) | <0.001 | 2.49 (2.35-2.63) | <0.001 |
| **Cholelithiasis** | 2.39 (2.18-2.63) | <0.001 | 1.60 (1.52-1.68) | <0.001 | 2.37 (2.27-2.47) | <0.001 |
| **Cholangitis** | 2.51 (1.94-3.24) | <0.001 | 1.42 (1.22-1.64) | <0.001 | 1.69 (1.47-1.93) | <0.001 |
| **ALD** | 2.45 (1.84-3.25) | <0.001 | 0.82 (0.69-0.98) | 0.026 | 1.50 (1.27-1.79) | <0.001 |
| **NAFLD** | 3.68 (3.18-4.27) | <0.001 | 1.68 (1.53-1.83) | <0.001 | 3.87 (3.57-4.18) | <0.001 |
| **Fibrosis and Cirrhosis of Liver** | 2.60 (2.04-3.32) | <0.001 | 0.79 (0.68-0.93) | 0.004 | 2.00 (1.75-2.29) | <0.001 |
| **Hepatic Failure** | 2.44 (1.73-3.46) | <0.001 | 0.99 (0.81-1.22) | 0.927 | 1.53 (1.25-1.87) | <0.001 |
| **GERD** | 1.39 (1.30-1.48) | <0.001 | 1.19 (1.15-1.23) | <0.001 | 1.37 (1.33-1.41) | <0.001 |
| **IBS** | 1.13 (0.98-1.30) | 0.082 | 0.90 (0.84-0.96) | 0.002 | 1.09 (1.03-1.15) | 0.002 |
| **Dyspepsia** | 0.98 (0.84-1.13) | 0.743 | 0.95 (0.89-1.02) | 0.142 | 0.93 (0.88-0.99) | 0.016 |
| **Functional Intestinal Disorders** | 1.22 (1.13-1.33) | <0.001 | 0.86 (0.82-0.89) | <0.001 | 1.05 (1.01-1.09) | 0.008 |
| **Oesophagitis** | 1.37 (1.20-1.57) | <0.001 | 1.12 (1.05-1.19) | <0.001 | 1.17 (1.10-1.24) | <0.001 |
| **Other Oesophagus Diseases** | 1.52 (1.37-1.69) | <0.001 | 1.13 (1.07-1.19) | <0.001 | 1.14 (1.08-1.19) | <0.001 |
| **Other Non Infective Gastroenteritis and Colitis** | 1.50 (1.35-1.66) | <0.001 | 0.95 (0.90-1.00) | 0.054 | 1.09 (1.04-1.15) | <0.001 |
| **Anus and Rectum Diseases** | 1.26 (1.16-1.38) | <0.001 | 1.04 (1.00-1.08) | 0.044 | 1.16 (1.11-1.20) | <0.001 |
| **Haemorrhoids** | 1.03 (0.93-1.14) | 0.593 | 0.97 (0.93-1.01) | 0.203 | 1.08 (1.04-1.13) | <0.001 |
| **GI Diseases Excluding Cancer** | 1.34 (1.29-1.39) | <0.001 | 1.06 (1.05-1.08) | <0.001 | 1.19 (1.17-1.20) | <0.001 |
| **GI Diseases** | 1.33 (1.28-1.39) | <0.001 | 1.06 (1.04-1.08) | <0.001 | 1.19 (1.17-1.20) | <0.001 |

**Table S7.** Sensitivity analyses for the association between clusters and risk of digestive diseases when excluding individuals diagnosed within two years of recruitment

| **Diseases** | **Inflammatory Status** | | **Overweight with high strength** | | **Obesity with Insulin Resistant** | |
| --- | --- | --- | --- | --- | --- | --- |
|  | **HR 95%CI** | ***P* value** | **HR 95%CI** | ***P* value** | **HR 95%CI** | ***P* value** |
| **Oesophagus Cancer** | 1.77 (1.22-2.57) | 0.003 | 1.05 (0.87-1.26) | 0.627 | 1.35 (1.11-1.64) | 0.002 |
| **Stomach Cancer** | 1.43 (0.84-2.41) | 0.184 | 1.26 (1.00-1.59) | 0.046 | 1.53 (1.22-1.92) | <0.001 |
| **Smallintestine Cancer** | 0.77 (0.28-2.11) | 0.612 | 0.83 (0.56-1.23) | 0.347 | 1.19 (0.84-1.69) | 0.316 |
| **Colorectal Cancer** | 1.38 (1.16-1.64) | <0.001 | 1.03 (0.95-1.11) | 0.496 | 1.17 (1.08-1.26) | <0.001 |
| **Anus Cancer** | 1.89 (0.87-4.11) | 0.107 | 0.83 (0.51-1.36) | 0.459 | 0.94 (0.61-1.44) | 0.773 |
| **Liver Cancer** | 1.60 (1.00-2.54) | 0.048 | 0.58 (0.45-0.75) | <0.001 | 1.43 (1.14-1.79) | 0.002 |
| **Gallbladder Cancer** | 1.23 (0.29-5.18) | 0.774 | 1.39 (0.68-2.87) | 0.368 | 2.42 (1.48-3.96) | <0.001 |
| **Biliarytract Cancer** | 1.27 (0.45-3.59) | 0.647 | 1.03 (0.65-1.65) | 0.899 | 1.32 (0.86-2.02) | 0.203 |
| **Pancreas Cancer** | 1.27 (0.86-1.88) | 0.224 | 1.21 (1.02-1.44) | 0.03 | 1.35 (1.15-1.59) | <0.001 |
| **GI Cancer** | 1.40 (1.23-1.60) | <0.001 | 1.02 (0.96-1.09) | 0.506 | 1.26 (1.18-1.33) | <0.001 |
| **IBD** | 2.45 (2.04-2.93) | <0.001 | 1.04 (0.94-1.16) | 0.461 | 1.08 (0.97-1.19) | 0.162 |
| **Gastritis and Duodenitis** | 1.20 (1.11-1.29) | <0.001 | 0.98 (0.94-1.01) | 0.19 | 1.09 (1.05-1.12) | <0.001 |
| **Gastritis** | 1.23 (1.13-1.33) | <0.001 | 0.95 (0.91-0.98) | 0.005 | 1.07 (1.03-1.11) | <0.001 |
| **Duodenitis** | 1.26 (1.09-1.46) | 0.002 | 1.09 (1.02-1.16) | 0.008 | 1.16 (1.09-1.24) | <0.001 |
| **Acute Pancreatitis** | 1.88 (1.46-2.44) | <0.001 | 1.29 (1.13-1.47) | <0.001 | 2.09 (1.86-2.34) | <0.001 |
| **Appendicitis** | 0.90 (0.64-1.26) | 0.541 | 1.07 (0.95-1.21) | 0.239 | 1.11 (0.99-1.25) | 0.075 |
| **Cholecystitis** | 2.39 (2.10-2.72) | <0.001 | 1.66 (1.55-1.78) | <0.001 | 2.47 (2.32-2.62) | <0.001 |
| **Cholelithiasis** | 2.33 (2.11-2.58) | <0.001 | 1.60 (1.51-1.69) | <0.001 | 2.34 (2.24-2.45) | <0.001 |
| **Cholangitis** | 2.47 (1.89-3.22) | <0.001 | 1.46 (1.26-1.70) | <0.001 | 1.70 (1.48-1.95) | <0.001 |
| **ALD** | 2.31 (1.70-3.15) | <0.001 | 0.88 (0.73-1.06) | 0.181 | 1.66 (1.38-1.98) | <0.001 |
| **NAFLD** | 3.66 (3.15-4.26) | <0.001 | 1.69 (1.54-1.86) | <0.001 | 3.85 (3.56-4.17) | <0.001 |
| **Fibrosis and Cirrhosis of Liver** | 2.77 (2.16-3.55) | <0.001 | 0.82 (0.70-0.97) | 0.019 | 2.08 (1.81-2.39) | <0.001 |
| **Hepatic Failure** | 2.20 (1.51-3.21) | <0.001 | 1.03 (0.83-1.27) | 0.806 | 1.53 (1.24-1.88) | <0.001 |
| **GERD** | 1.40 (1.30-1.50) | <0.001 | 1.19 (1.16-1.23) | <0.001 | 1.36 (1.33-1.40) | <0.001 |
| **IBS** | 1.11 (0.96-1.29) | 0.149 | 0.91 (0.85-0.98) | 0.008 | 1.09 (1.03-1.15) | 0.005 |
| **Dyspepsia** | 0.97 (0.82-1.15) | 0.738 | 0.96 (0.90-1.04) | 0.326 | 0.93 (0.87-1.00) | 0.041 |
| **Functional Intestinal Disorders** | 1.17 (1.07-1.27) | <0.001 | 0.86 (0.83-0.90) | <0.001 | 1.07 (1.03-1.11) | <0.001 |
| **Oesophagitis** | 1.36 (1.18-1.57) | <0.001 | 1.10 (1.03-1.18) | 0.003 | 1.15 (1.08-1.23) | <0.001 |
| **Other Oesophagus Diseases** | 1.49 (1.34-1.67) | <0.001 | 1.13 (1.07-1.19) | <0.001 | 1.14 (1.08-1.20) | <0.001 |
| **Other Non Infective Gastroenteritis and Colitis** | 1.39 (1.23-1.57) | <0.001 | 0.96 (0.91-1.02) | 0.197 | 1.07 (1.01-1.13) | 0.013 |
| **Anus and Rectum Diseases** | 1.21 (1.09-1.33) | <0.001 | 1.04 (1.00-1.08) | 0.067 | 1.15 (1.11-1.20) | <0.001 |
| **Haemorrhoids** | 1.03 (0.93-1.14) | 0.601 | 0.98 (0.94-1.02) | 0.236 | 1.09 (1.04-1.13) | <0.001 |
| **GI Diseases Excluding Cancer** | 1.30 (1.24-1.35) | <0.001 | 1.07 (1.05-1.09) | <0.001 | 1.19 (1.17-1.21) | <0.001 |
| **GI Diseases** | 1.29 (1.24-1.35) | <0.001 | 1.07 (1.05-1.09) | <0.001 | 1.19 (1.17-1.21) | <0.001 |

**Table S8.** Sensitivity analyses for the association between clusters and risk of digestive diseases when excluding those with less than two years of follow-up

| **Diseases** | **Inflammatory Status** | | **Overweight with high strength** | | **Obesity with Insulin Resistant** | |
| --- | --- | --- | --- | --- | --- | --- |
|  | **HR 95%CI** | ***P* value** | **HR 95%CI** | ***P* value** | **HR 95%CI** | ***P* value** |
| **Oesophagus Cancer** | 1.77 (1.22-2.57) | 0.003 | 1.05 (0.87-1.26) | 0.627 | 1.35 (1.11-1.64) | 0.002 |
| **Stomach Cancer** | 1.43 (0.84-2.41) | 0.184 | 1.26 (1.00-1.59) | 0.046 | 1.53 (1.22-1.92) | <0.001 |
| **Smallintestine Cancer** | 0.77 (0.28-2.11) | 0.612 | 0.83 (0.56-1.23) | 0.347 | 1.19 (0.84-1.69) | 0.316 |
| **Colorectal Cancer** | 1.38 (1.16-1.64) | <0.001 | 1.03 (0.95-1.11) | 0.502 | 1.16 (1.08-1.26) | <0.001 |
| **Anus Cancer** | 1.88 (0.87-4.08) | 0.111 | 0.85 (0.52-1.38) | 0.504 | 0.94 (0.61-1.43) | 0.759 |
| **Liver Cancer** | 1.60 (1.00-2.54) | 0.048 | 0.58 (0.45-0.75) | <0.001 | 1.43 (1.14-1.79) | 0.002 |
| **Gallbladder Cancer** | 1.23 (0.29-5.18) | 0.774 | 1.39 (0.68-2.87) | 0.368 | 2.42 (1.48-3.96) | <0.001 |
| **Biliarytract Cancer** | 1.27 (0.45-3.59) | 0.647 | 1.03 (0.65-1.65) | 0.899 | 1.32 (0.86-2.02) | 0.203 |
| **Pancreas Cancer** | 1.27 (0.86-1.88) | 0.224 | 1.21 (1.02-1.44) | 0.03 | 1.35 (1.15-1.59) | <0.001 |
| **GI Cancer** | 1.40 (1.23-1.60) | <0.001 | 1.02 (0.96-1.09) | 0.505 | 1.26 (1.18-1.33) | <0.001 |
| **IBD** | 2.45 (2.04-2.93) | <0.001 | 1.04 (0.94-1.16) | 0.461 | 1.08 (0.97-1.19) | 0.162 |
| **Gastritis and Duodenitis** | 1.20 (1.11-1.29) | <0.001 | 0.98 (0.94-1.01) | 0.192 | 1.09 (1.05-1.12) | <0.001 |
| **Gastritis** | 1.23 (1.14-1.33) | <0.001 | 0.95 (0.91-0.98) | 0.005 | 1.07 (1.03-1.11) | <0.001 |
| **Duodenitis** | 1.26 (1.09-1.46) | 0.002 | 1.09 (1.02-1.16) | 0.008 | 1.16 (1.09-1.24) | <0.001 |
| **Acute Pancreatitis** | 1.88 (1.45-2.44) | <0.001 | 1.29 (1.13-1.47) | <0.001 | 2.09 (1.86-2.34) | <0.001 |
| **Appendicitis** | 0.90 (0.64-1.26) | 0.541 | 1.07 (0.95-1.21) | 0.239 | 1.11 (0.99-1.25) | 0.075 |
| **Cholecystitis** | 2.39 (2.10-2.72) | <0.001 | 1.66 (1.55-1.78) | <0.001 | 2.47 (2.33-2.62) | <0.001 |
| **Cholelithiasis** | 2.33 (2.11-2.58) | <0.001 | 1.60 (1.51-1.69) | <0.001 | 2.34 (2.24-2.46) | <0.001 |
| **Cholangitis** | 2.47 (1.89-3.22) | <0.001 | 1.46 (1.26-1.70) | <0.001 | 1.70 (1.48-1.95) | <0.001 |
| **ALD** | 2.31 (1.70-3.15) | <0.001 | 0.88 (0.73-1.06) | 0.181 | 1.66 (1.38-1.98) | <0.001 |
| **NAFLD** | 3.66 (3.15-4.26) | <0.001 | 1.69 (1.54-1.85) | <0.001 | 3.85 (3.56-4.17) | <0.001 |
| **Fibrosis and Cirrhosis of Liver** | 2.77 (2.16-3.55) | <0.001 | 0.82 (0.70-0.97) | 0.019 | 2.08 (1.81-2.39) | <0.001 |
| **Hepatic Failure** | 2.20 (1.51-3.21) | <0.001 | 1.03 (0.83-1.27) | 0.806 | 1.53 (1.24-1.88) | <0.001 |
| **GERD** | 1.40 (1.30-1.50) | <0.001 | 1.19 (1.16-1.23) | <0.001 | 1.36 (1.33-1.40) | <0.001 |
| **IBS** | 1.11 (0.96-1.29) | 0.15 | 0.91 (0.85-0.98) | 0.008 | 1.09 (1.03-1.15) | 0.005 |
| **Dyspepsia** | 0.97 (0.82-1.15) | 0.738 | 0.96 (0.90-1.04) | 0.326 | 0.93 (0.87-1.00) | 0.041 |
| **Functional Intestinal Disorders** | 1.17 (1.07-1.27) | <0.001 | 0.86 (0.83-0.90) | <0.001 | 1.07 (1.03-1.11) | <0.001 |
| **Oesophagitis** | 1.36 (1.18-1.57) | <0.001 | 1.10 (1.03-1.18) | 0.004 | 1.15 (1.08-1.23) | <0.001 |
| **Other Oesophagus Diseases** | 1.49 (1.34-1.67) | <0.001 | 1.13 (1.07-1.19) | <0.001 | 1.14 (1.08-1.20) | <0.001 |
| **Other Non Infective Gastroenteritis and Colitis** | 1.39 (1.23-1.57) | <0.001 | 0.96 (0.91-1.02) | 0.201 | 1.07 (1.01-1.13) | 0.013 |
| **Anus and Rectum Diseases** | 1.20 (1.09-1.33) | <0.001 | 1.04 (1.00-1.08) | 0.067 | 1.15 (1.11-1.20) | <0.001 |
| **Haemorrhoids** | 1.03 (0.93-1.14) | 0.601 | 0.98 (0.94-1.02) | 0.236 | 1.09 (1.04-1.13) | <0.001 |
| **GI Diseases Excluding Cancer** | 1.30 (1.24-1.35) | <0.001 | 1.07 (1.05-1.09) | <0.001 | 1.19 (1.17-1.21) | <0.001 |
| **GI Diseases** | 1.29 (1.24-1.35) | <0.001 | 1.07 (1.05-1.09) | <0.001 | 1.19 (1.17-1.21) | <0.001 |

**Table S9.** Mediation analysis of metabolic signature between Inflammatory Status and Healthy Status with Low Strength in relation to digestive diseases. Adjusted for age, age squared, sex, ethnicity, Townsend deprivation index, smoking status, alcohol drinking, education level, physical activity, medicinal intake (aspirin, non-aspirin NSAIDs, and lipid-lowering drugs use), and comorbidities (lipidaemia, hypertension, diabetes).

| **Diseases** | **Total effect** | ***P* value** | **Nature direct effect** | ***P* value** | **Nature indirect effect** | ***P* value** | **Mediated Proportion** | ***P* value** |
| --- | --- | --- | --- | --- | --- | --- | --- | --- |
| GI Cancer | 1.50 (1.25-1.75) | **<0.001** | 1.64 (1.38-1.92) | **<0.001** | 0.91 (0.86-0.98) | **0.010** | 28% | **0.010** |
| GERD | 1.34 (1.23-1.46) | **<0.001** | 1.62 (1.48-1.76) | **<0.001** | 0.83 (0.80-0.86) | **<0.001** | 81% | **<0.001** |
| Other Oesophagus Diseases | 1.55 (1.34-1.74) | **<0.001** | 1.91 (1.65-2.20) | **<0.001** | 0.81 (0.77-0.86) | **<0.001** | 67% | **<0.001** |
| Gastritis and Duodenitis | 1.23 (1.09-1.34) | **<0.001** | 1.41 (1.26-1.56) | **<0.001** | 0.87 (0.84-0.90) | **<0.001** | 82% | **<0.001** |
| IBD | 2.29 (1.80-2.95) | **<0.001** | 3.40 (2.66-4.34) | **<0.001** | 0.67 (0.61-0.75) | **<0.001** | 87% | **<0.001** |
| Gastritis | 1.25 (1.13-1.38) | **<0.001** | 1.44 (1.29-1.60) | **<0.001** | 0.87 (0.84-0.90) | **<0.001** | 75% | **<0.001** |
| Duodenitis | 1.35 (1.12-1.63) | **<0.001** | 1.63 (1.31-1.99) | **<0.001** | 0.83 (0.78-0.89) | **<0.001** | 78% | **<0.001** |
| Other Non-Infective Gastroenteritis and Colitis | 1.45 (1.24-1.68) | **<0.001** | 1.57 (1.33-1.83) | **<0.001** | 0.92 (0.88-0.98) | **0.005** | 26% | **0.005** |
| ALD | 2.83 (1.75-4.51) | **<0.001** | 4.66 (2.79-7.96) | **<0.001** | 0.61 (0.49-0.74) | **<0.001** | 99% | **<0.001** |
| Hepatic Failure | 3.02 (1.97-4.39) | **<0.001** | 4.55 (2.85-6.96) | **<0.001** | 0.66 (0.53-0.84) | **0.005** | 76% | **0.005** |
| NAFLD | 4.32 (3.55-5.19) | **<0.001** | 4.76 (3.92-5.90) | **<0.001** | 0.91 (0.83-1.00) | **0.040** | 13% | **0.040** |
| Cholecystitis | 2.37 (2.00-2.81) | **<0.001** | 3.09 (2.62-3.64) | **<0.001** | 0.77 (0.72-0.82) | **<0.001** | 53% | **<0.001** |
| Cholelithiasis | 2.24 (1.96-2.53) | **<0.001** | 2.80 (2.46-3.19) | **<0.001** | 0.80 (0.76-0.84) | **<0.001** | 45% | **<0.001** |
| Cholangitis | 2.95 (2.00-4.20) | **<0.001** | 3.98 (2.87-5.62) | **<0.001** | 0.74 (0.63-0.86) | **<0.001** | 53% | **<0.001** |
| Acute Pancreatitis | 2.09 (1.49-2.78) | **<0.001** | 2.63 (1.84-3.51) | **<0.001** | 0.79 (0.71-0.91) | **<0.001** | 50% | **<0.001** |
| GI Diseases Excluding Cancer | 1.34 (1.26-1.40) | **<0.001** | 1.45 (1.38-1.53) | **<0.001** | 0.92 (0.91-0.94) | **<0.001** | 35% | **<0.001** |
| GI Diseases | 1.33 (1.25-1.40) | **<0.001** | 1.45 (1.36-1.53) | **<0.001** | 0.92 (0.91-0.93) | **<0.001** | 35% | **<0.001** |

**Table S10.** Mediation analysis of metabolic signature between Overweight with High Strength and Healthy Status with Low Strength in relation to digestive diseases. Adjusted for age, age squared, sex, ethnicity, Townsend deprivation index, smoking status, alcohol drinking, education level, physical activity, medicinal intake (aspirin, non-aspirin NSAIDs, and lipid-lowering drugs use), and comorbidities (lipidaemia, hypertension, diabetes).

| **Diseases** | **Total effect** | ***P* value** | **Nature direct effect** | ***P* value** | **Nature indirect effect** | ***P* value** | **Mediated Proportion** | ***P* value** |
| --- | --- | --- | --- | --- | --- | --- | --- | --- |
| Oesophagitis | 1.10 (1.01-1.19) | **0.025** | 1.03 (0.95-1.11) | 0.405 | 1.06 (1.05-1.08) | **<0.001** | 66% | **0.025** |
| GERD | 1.16 (1.12-1.22) | **<0.001** | 1.12 (1.07-1.17) | **<0.001** | 1.04 (1.04-1.05) | **<0.001** | 29% | **<0.001** |
| Other Oesophagus Diseases | 1.14 (1.07-1.23) | **<0.001** | 1.09 (1.02-1.17) | **0.005** | 1.05 (1.04-1.06) | **<0.001** | 37% | **<0.001** |
| Gastritis | 0.93 (0.89-0.97) | **0.005** | 0.90 (0.86-0.94) | **<0.001** | 1.03 (1.02-1.04) | **<0.001** | 43% | **0.005** |
| Duodenitis | 1.10 (1.01-1.20) | **0.019** | 1.05 (0.98-1.15) | 0.205 | 1.04 (1.03-1.06) | **<0.001** | 46% | **0.010** |
| NAFLD | 1.68 (1.49-1.89) | **<0.001** | 1.64 (1.46-1.85) | **<0.001** | 1.02 (1.00-1.04) | **0.025** | 5% | **0.025** |
| Cholecystitis | 1.54 (1.40-1.70) | **<0.001** | 1.45 (1.32-1.60) | **<0.001** | 1.06 (1.05-1.07) | **<0.001** | 17% | **<0.001** |
| Cholelithiasis | 1.48 (1.38-1.57) | **<0.001** | 1.41 (1.31-1.50) | **<0.001** | 1.05 (1.04-1.06) | **<0.001** | 15% | **<0.001** |
| Cholangitis | 1.54 (1.25-1.90) | **<0.001** | 1.44 (1.16-1.82) | **<0.001** | 1.07 (1.03-1.11) | **<0.001** | 19% | **<0.001** |
| Acute Pancreatitis | 1.27 (1.10-1.50) | **0.005** | 1.21 (1.03-1.42) | **0.039** | 1.05 (1.03-1.09) | **<0.001** | 24% | **0.005** |
| GI Diseases Excluding Cancer | 1.06 (1.03-1.08) | **<0.001** | 1.03 (1.01-1.06) | **<0.001** | 1.02 (1.02-1.03) | **<0.001** | 40% | **<0.001** |
| GI Diseases | 1.06 (1.03-1.08) | **<0.001** | 1.03 (1.01-1.06) | **0.005** | 1.02 (1.02-1.03) | **<0.001** | 41% | **<0.001** |

**Table S11.** Mediation analysis of metabolic signature between Obesity with Insulin Resistant and Healthy Status with Low Strength in relation to digestive diseases. Adjusted for age, age squared, sex, ethnicity, Townsend deprivation index, smoking status, alcohol drinking, education level, physical activity, medicinal intake (aspirin, non-aspirin NSAIDs, and lipid-lowering drugs use), and comorbidities (lipidaemia, hypertension, diabetes).

| **Diseases** | **Total effect** | ***P* value** | **Nature direct effect** | ***P* value** | **Nature indirect effect** | ***P* value** | **Mediated Proportion** | ***P* value** |
| --- | --- | --- | --- | --- | --- | --- | --- | --- |
| GI Cancer | 1.26 (1.17-1.37) | **<0.001** | 1.25 (1.16-1.35) | **<0.001** | 1.01 (1.00-1.02) | **0.005** | 6% | **0.005** |
| Oesophagitis | 1.15 (1.05-1.26) | **0.010** | 1.11 (1.01-1.20) | **0.040** | 1.04 (1.03-1.05) | **<0.001** | 29% | **0.010** |
| GERD | 1.37 (1.32-1.42) | **<0.001** | 1.33 (1.28-1.39) | **<0.001** | 1.03 (1.02-1.03) | **<0.001** | 10% | **<0.001** |
| Other Oesophagus Diseases | 1.18 (1.10-1.26) | **<0.001** | 1.14 (1.07-1.22) | **<0.001** | 1.03 (1.02-1.04) | **<0.001** | 20% | **<0.001** |
| Gastritis and Duodenitis | 1.10 (1.06-1.15) | **<0.001** | 1.07 (1.03-1.12) | **<0.001** | 1.02 (1.02-1.03) | **<0.001** | 24% | **<0.001** |
| Gastritis | 1.08 (1.03-1.13) | **<0.001** | 1.06 (1.01-1.11) | **0.01** | 1.02 (1.02-1.03) | **<0.001** | 29% | **<0.001** |
| Duodenitis | 1.18 (1.09-1.29) | **<0.001** | 1.15 (1.06-1.26) | **<0.001** | 1.03 (1.02-1.04) | **<0.001** | 17% | **<0.001** |
| Other Non-Infective Gastroenteritis and Colitis | 1.10 (1.03-1.18) | **0.005** | 1.09 (1.02-1.17) | **0.025** | 1.01 (1.00-1.02) | **<0.001** | 12% | **0.005** |
| ALD | 2.26 (1.72-3.14) | **<0.001** | 2.11 (1.61-2.86) | **<0.001** | 1.07 (1.04-1.10) | **<0.001** | 12% | **<0.001** |
| Hepatic Failure | 1.66 (1.26-2.17) | **<0.001** | 1.57 (1.21-2.04) | **<0.001** | 1.06 (1.02-1.10) | **<0.001** | 14% | **<0.001** |
| NAFLD | 3.81 (3.40-4.23) | **<0.001** | 3.75 (3.33-4.17) | **<0.001** | 1.01 (1.00-1.03) | **0.025** | 2% | **0.025** |
| Cholecystitis | 2.39 (2.18-2.58) | **<0.001** | 2.30 (2.10-2.48) | **<0.001** | 1.04 (1.03-1.05) | **<0.001** | 6% | **<0.001** |
| Cholelithiasis | 2.32 (2.17-2.47) | **<0.001** | 2.24 (2.09-2.40) | **<0.001** | 1.03 (1.02-1.04) | **<0.001** | 6% | **<0.001** |
| Cholangitis | 1.84 (1.56-2.24) | **<0.001** | 1.77 (1.49-2.14) | **<0.001** | 1.04 (1.02-1.06) | **<0.001** | 9% | **<0.001** |
| Acute Pancreatitis | 2.02 (1.75-2.32) | **<0.001** | 1.96 (1.70-2.25) | **<0.001** | 1.03 (1.01-1.05) | **<0.001** | 6% | **<0.001** |
| GI Diseases Excluding Cancer | 1.19 (1.17-1.22) | **<0.001** | 1.18 (1.15-1.20) | **<0.001** | 1.02 (1.01-1.02) | **<0.001** | 10% | **<0.001** |
| GI Diseases | 1.19 (1.17-1.22) | **<0.001** | 1.17 (1.15-1.20) | **<0.001** | 1.02 (1.01-1.02) | **<0.001** | 10% | **<0.001** |

**Table S12.** ICD-10 Codes for Digestive Diseases

| Category | Disease Name | ICD-10 Code(s) |
| --- | --- | --- |
| Digestive Cancers | Oesophagus Cancer | C15 |
|  | Stomach Cancer | C16 |
|  | Small Intestine Cancer | C17 |
|  | Colorectal Cancer | C18–C20 |
|  | Anus Cancer | C21 |
|  | Liver Cancer | C22 |
|  | Gallbladder Cancer | C23 |
|  | Biliary Tract Cancer | C24 |
|  | Pancreas Cancer | C25 |
| Immune/Inflammatory Disorders | Inflammatory Bowel Disease (IBD) | K50, K51 |
|  | Gastritis and Duodenitis | K29 |
|  | Gastritis | K29.3–K29.7 |
|  | Duodenitis | K29.8 |
|  | Acute Pancreatitis | K85 |
|  | Appendicitis | K35–K37 |
|  | Cholecystitis | K81 |
|  | Cholangitis | K83.0 |
| Metabolic Diseases | Alcoholic Liver Disease (ALD) | K70 |
|  | Non-Alcoholic Fatty Liver Disease (NAFLD) | K76.0 |
|  | Fibrosis and Cirrhosis of Liver | K74 |
|  | Hepatic Failure | K72 |
| Functional Disorders | Gastro-oesophageal Reflux Disease (GERD) | K21 |
|  | Irritable Bowel Syndrome (IBS) | K58 |
|  | Dyspepsia | K30 |
|  | Functional Intestinal Disorders | K59 |
|  | Oesophagitis | K20 |
|  | Other Oesophagus Diseases | K22 |
|  | Other Non-infective Gastroenteritis/Colitis | K52 |
|  | Anus and Rectum Diseases | K62 |
|  | Haemorrhoids | I84 |

**Table S13.** Proportion of missing data for six parameters in the study

| Parameters | Missing Data (Missing/Total) | Missing Data Proportion (%) |
| --- | --- | --- |
| BMI | 3103/502173 | 0.62% |
| WHtR | 2677/502173 | 0.53% |
| Grip strength | 2360/502173 | 0.47% |
| CRP | 33909/502173 | 6.75% |
| NLR | 25201/502173 | 5.02% |
| TyG-BMI | 74939/502173 | 14.92% |

Abbreviations: BMI, Body Mass Index; WHtR, Waist-to-Height Ratio; CRP, C-Reactive Protein; NLR, Neutrophil-to-Lymphocyte Ratio; TyG-BMI, triglyceride-glucose index-BMI.
